# Forest Filter Effect Revisited: First Evidence That Polycyclic Aromatic Hydrocarbon Metabolites Are Produced on Leaves by Biodegradation and Photodegradation

**DOI:** 10.1021/acs.est.5c09252

**Published:** 2025-09-15

**Authors:** Elisa Terzaghi, Corinne Bertipaglia, Elisabetta Zanardini, Davide Siniscalchi, Renzo Bagnati, Alice Passoni, Laura Rampazzi, Cristina Corti, Josè-Julio Ortega-Calvo, Rosa Posada-Baquero, Antonio Di Guardo

**Affiliations:** † Department of Science and High Technology, University of Insubria, Via Valleggio 11, 22100 Como, Italy; ‡ Department of Human Sciences and Innovation for the Territory, University of Insubria, Via Sant’Abbondio 12, 22100 Como, Italy; § Instituto de Recursos Naturales y Agrobiologıá de Sevilla (IRNAS-CSIC), Avda. Reina Mercedes, 10, E-41080 Seville, Spain; ∥ Department of Environmental Health Sciences, Istituto di Ricerche Farmacologiche “Mario Negri” IRCCS, Via Mario Negri 2, 20156 Milan, Italy

**Keywords:** PAH metabolites, ecosystem services, phyllosphere, suspect screening analyses, air quality improvement, biodegradation, photodegradation

## Abstract

Plants are considered “nature-based solutions” for the improvement of air quality. Through the so-called forest filter effect (FFE), they can remove both gas and particulate matter (PM) associated organic contaminants, such as polycyclic aromatic hydrocarbons (PAHs) from air, reducing their concentrations. In this work, new aspects of FFE were investigated through several laboratory experiments of increasing complexity. More specifically, the study was designed to evaluate for the first time the contribution of light and phyllosphere microbial communities of *Quercus ilex* leaves to the degradation of deuterated PAHs (naphthalene-d8, acenaphthene-d10, phenanthrene-d10, pyrene-d10, chrysene-d12, and perylene-d12). Such degradation was investigated by observing the production of deuterated PAH hydroxy metabolites (OH-PAHs). OH-PAHs were produced in both light and dark conditions, while their appearance in sterile controls was negligible, highlighting the importance of leaf-associated microorganisms in the degradation of parent PAH. The number of PAH metabolites and their isomers increased when entire leaves were used rather than the inoculated phyllosphere microorganisms only, confirming the degradation ability of native phyllosphere microbial communities. Although preliminary, these results showed that FFE can be enhanced by photo- and biodegradation processes. The continuous removal of PAHs through degradation could result in an enhanced PAH concentration gradient and flux of deposition to leaves.

## Graphical abstract

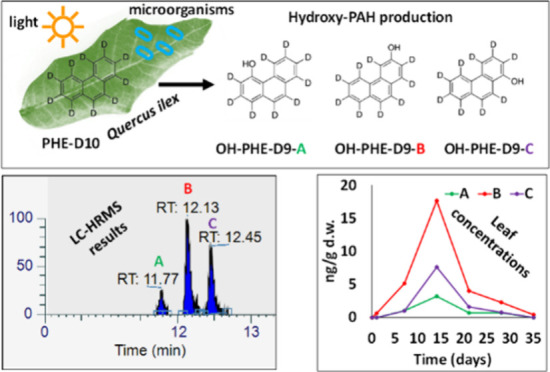

## Introduction

Polycyclic aromatic hydrocarbons (PAHs) are ubiquitous environmental organic contaminants that originate mainly from anthropogenic sources, e.g., industrial processes, vehicular traffic, domestic heating, etc., through the incomplete combustion of organic materials. Once emitted from urban and rural sites, they can partition between the air gas phase and particulate matter (PM) and undergo long-range transport, affecting also remote areas.^1,2^ In the last few decades, the scientific evidence of their toxicological^3^ and ecotoxicological^4^ properties led to the adoption of many policy instruments to monitor and regulate PAH air concentrations in urban areas.^5,6^ Emission reduction, fossil fuel substitution with renewable energies, and remediation strategies could jointly contribute to air quality improvement.^7^

An additional way to improve urban air quality could be reached by increasing forested areas. In fact, trees can be seen as “nature-based solutions” (NBS) which could provide important ecosystem services (ES),^8^ and among these, reduce air pollution.^9^ Plants are indeed well known to act as filter of both gas- and PM-associated PAHs^10,11^ (e.g., forest filter effect (FFE)). Plant leaves can be seen as chemical and biological reactors, where photo- and biodegradation of organic contaminants may occur. This is due to the larger foliar surface area than the corresponding soil area, where they live. Photodegradation on leaves was shown to be an important loss mechanism for PAHs,^12^ but it was also shown in recent studies that the phyllosphere can host microorganisms capable of biodegrading atmospheric PAH.^13,14^ Terzaghi et al.^15^ first modeled the influence of photo- and biodegradation of phenanthrene and benzo­[a]­pyrene leaf concentrations of urban trees in the towns of Como and Naples, Italy. It was shown that leaf PAH concentrations could be reduced of more than 25%, together with their potential re-emission fluxes toward air and soil. Recently, it was also found that PM could modulate the bioaccessibility of pyrene on leaf surfaces, therefore speeding up microbial degradation.^16^ However, photo- and biodegradation studies for PAHs on leaves are still scarce,^12,17–23^ especially those investigating PAH mineralization^16^ and PAH metabolite production,^17,24^ rather than parent compound disappearance. This highlights the need to better understand the potential interaction between light-mediated reactions and the activity of microbial communities in the phyllosphere for PAH removal from air (phylloremediation).

The purpose of this article was to obtain the first evidence of PAH degradation occurring on leaves, showing the production of PAH polar metabolites (i.e., hydroxy-PAHs), starting from deuterated PAHs. More specifically, this work investigated novel aspects of FFE, including light-mediated PAH degradation (i.e., photodegradation) and their biodegradation by phyllosphere microorganisms on holm oak (*Quercus ilex*) leaves, as model species, using deuterated standards as parent compounds. No efforts were made to furnish a full quantitative assessment of PAH metabolite production or degradation path but rather to illustrate the conditions and factors involved. This was performed through several microcosm experiments simulating different microbial growth conditions and leaf exposure. To the best of our knowledge, this is the first work investigating PAH hydroxy metabolite production on leaves.

## Methods and Materials

### Experimental Design

Photo- and biodegradation processes of deuterated PAH and PAH metabolite production were investigated in three different microcosms of increasing ecological complexity (Figure 1): “water contact system, phyllosphere microorganisms” microcosm (WCS-PhMi), “water contact system, entire leaf” microcosm (WCS-EL) and “air contact system, entire leaf” microcosm (ACS-EL). The WCS-PhMi system was designed to investigate the degradation of PAHs by phyllosphere microorganisms alone, obtained from holm oak leaves. The WCS-EL system was designed to evaluate the degradation activity of phyllosphere microbial communities growing directly in/on leaves immersed in mineral medium. The ACS-EL system was set up to study PAH degradation on leaves exposed to air. The WCS systems allowed us to investigate PAH metabolite production under a setup aimed at optimizing microbial activity. This was due to the renewal of nutrients, oxygen, and PAHs as carbon sources due to the agitation conditions. The ACS system allowed us to reproduce more realistic field-like conditions where leaves are in contact with air rather than water, under static conditions, like in Terzaghi et al.^16^ All systems included light, dark, and sterilized treatments as well as not spiked controls. Deuterated PAHs were spiked into each system, and their degradation activity was measured following the production of some PAH metabolites (i.e., mono- and dihydroxy-PAHs) rather than the disappearance of the parent compound. PAHs were selected for their environmental relevance and presence in air as well as for their increasing molecular weight and complexity, often related to their recalcitrance to biodegradation.^3^ The use of labeled PAHs allowed us to measure the production of PAH metabolites derived from the degradation of the spiked compounds rather than from those already present on leaves (i.e., native PAHs). More details about the experimental design are reported in the following sections and in Table 1.

**Figure 1:**
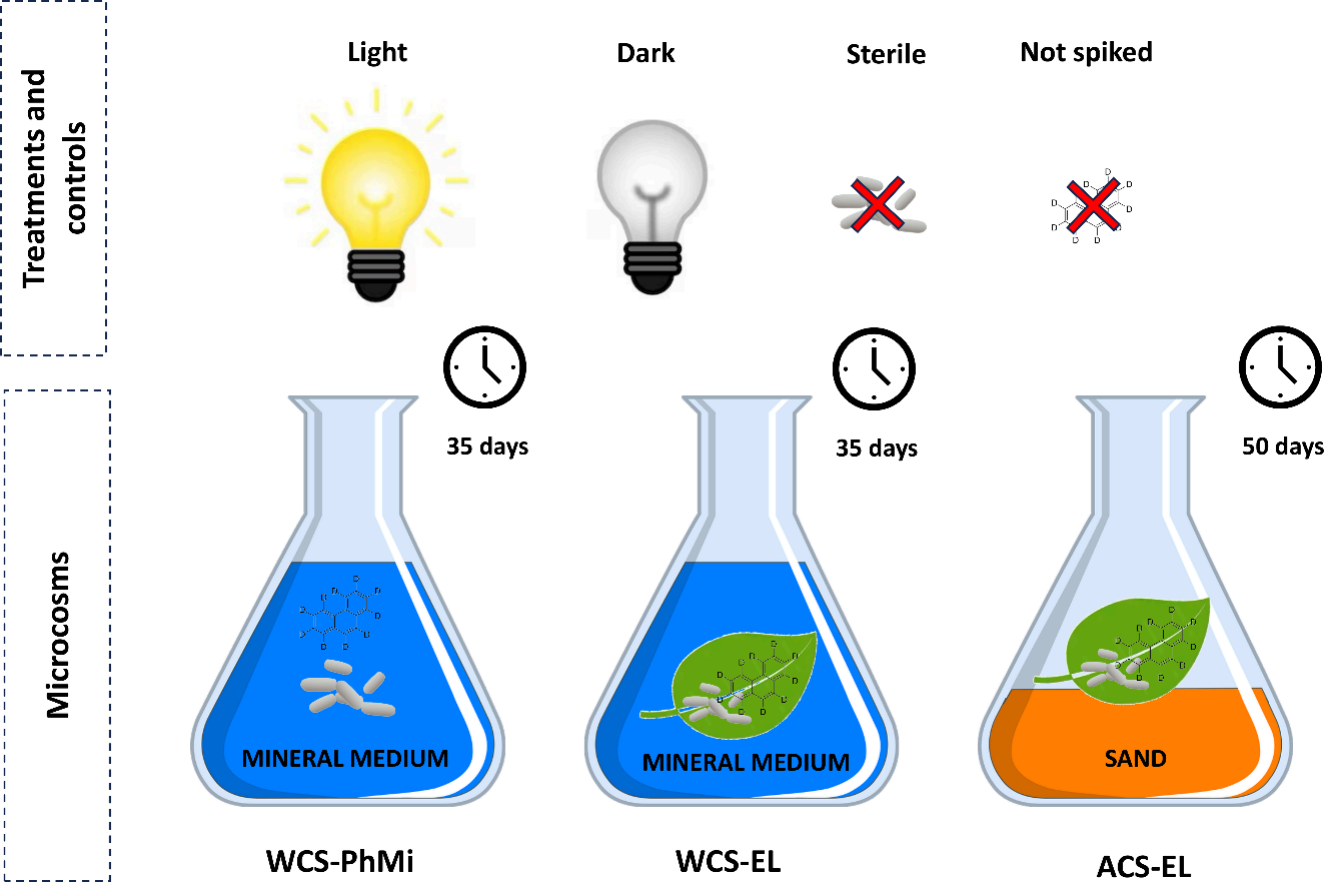
Schematic representation of the experimental design. Microcosm names are as follows: WCS-PhMi stands for “water contact system, phyllosphere microorganisms”, WCS–EL for “water contact system, entire leaf”, and ACS–EL for “air contact system, entire leaf”.

**Table 1:** Comparison of Experiment Conditions and Analyses^a^

	WCS-PhMi	WCS-EL	ACS-EL
Exposure media	100 mL mineral medium (MM)	200 mL mineral medium (MM)	40 g wet sand, air
Microbial load	10^6^ CFU/flask	10^6^ CFU/flask (10^5^–10^6^ CFU/g of fresh leaves)	(10^5^–10^6^ CFU/g of fresh leaves)
Leaves	No, just microorganisms	6 in each flask	2 in each flask
PAH spiked amount	800 ng of naphthalene-d8, acenaphthene-d10, phenanthrene-d10, chrysene-d12, perylene-d12, and 400 ng of pyrene-d10 in 100 mL MM	800 ng of naphthalene-d8, acenaphthene-d10, phenanthrene-d10, chrysene-d12, perylene-d12, and 400 ng of pyrene-d10 on each leaf	800 ng of naphthalene-d8, acenaphthene-d10, phenanthrene-d10, chrysene-d12, perylene-d12 on each leaf
Duration	35 days	35 days	50 days
Sampling time	0, 1, 7, 14, 21, 28, and 35 days	0, 1, 7, 14, 21, 28, and 35 days	0, 15, 50 days
Treatments	light, dark, sterile (no inoculum)	light, dark, sterile	light, dark, sterile
Controls	light not spiked	light not spiked	light not spiked
Chemical analyses	10 mL of MM for OH-PAH at each sampling time	1 leaf for OH-PAH at each sampling time	1 leaf for OH-PAH at each sampling time
Microbiological analyses	microbial counts and bacterial community structure at T0 and T35	microbial counts and bacterial community structure at T0 and T35	not available

a Microcosm names are as follows: WCS-PhMi stands for “water contact system, phyllosphere microorganisms”, WCS-EL for “water contact system, entire leaf”, and for ACS-EL “air contact system, entire leaf”.

### WCS-PhMi Experiment

One 250 mL flask for each treatment was filled with 100 mL of M9 mineral medium (Na_2_HPO_4_ 6 g/L, KH_2_PO_4_ 3 g/L, NH_4_Cl 1 g/L, NaCl 0.5 g/L, MgSO_4_ 0.12 g/L, CaCl_2_ 0.01 g/L) and spiked with a mix of deuterated PAHs: 800 ng of naphthalene-d8, acenaphthene-d10, phenanthrene-d10, chrysene-d12, perylene-d12, and 400 ng of pyrene-d10, dissolved in acetone (50 μL) as in Terzaghi et al.^16^ An inoculum of 10^4^ colony forming units or CFU/mL of phyllosphere microorganisms was added to each flask. The inoculum was obtained by washing holm oak leaves as described in the Microorganism Inoculum, Medium, and Cultivation section. Flasks were closed with vent cups and incubated for 35 days at room temperature on a shaker operating at 80 rpm and sampled at 0, 1, 7, 14, 21, 28, and 35 days and analyzed for deuterated OH-PAH and di-OH-PAH determination. The bacterial community was also analyzed in the last sample (i.e., 35 days). Three treatments were considered: flask exposed to light (“light flask”), flask shaded from light (“dark flask”), and flask exposed to light but without inoculum (“sterile flask”). Mineral medium sterility was obtained by filtration at 0.2 μm, while dry heat sterilization was used for glass flasks. Light and sterile flasks were exposed to the diurnal variability of natural light occurring in the laboratory (no specific lamps were used, the illuminance varied between 500 to 3000 lx), while the dark flask was covered with aluminum foil and black paper tape. A control flask without deuterated PAH was also set up.

### WCS-EL Experiment

Twenty-one leaves of holm oak were each spiked with the same amounts of the deuterated PAH mix dissolved in 50 μL of acetone After acetone evaporation, six leaves, for each treatment, were placed in a 250 mL flask with 200 mL of M9 mineral medium. One leaf was frozen immediately for further analyses representing the initial conditions (time 0 days). Similarly to the WCS-PhMi experiment, three treatments were considered: flask exposed to light (“light flask”), flask shaded from light (“dark flask”), and flask exposed to light but with sterile leaves (“sterile flask”). A control flask without deuterated PAH was also set up. Flasks were closed with a vent cap and agitated on a shaker operating at 80 rpm for 35 days at room temperature. One leaf was sampled from each flask at times of 1, 7, 14, 21, 28, and 35 days and analyzed for deuterated OH-PAH and di-OH-PAH determination. It was chosen to measure leaves instead of mineral medium for a number of reasons: metabolites, although more polar than the native compounds, would preferably and quantitatively partition to an organic phase (the leaf) rather than a water phase. Additionally, measuring metabolites in leaves would have allowed a comparison with native PAH metabolites found and produced in the same leaves. The bacterial community was also analyzed in the last samples (i.e., 35 days).

### ACS-EL Experiment

Forty grams of sterilized sand was placed in a 250 mL flask, with 15 mL of sterilized deionized water. Two leaves for each treatment were gently introduced into the flask over the wet sand and spiked with 800 ng each of deuterated PAH mix (naphthalene-d8, acenaphthene-d10, phenanthrene-d10, chrysene-d12, perylene-d12) dissolved in acetone (50 μL), as in Terzaghi et al.^16^ An additional leaf was spiked and frozen for further analyses representing the initial conditions (time of 0 days). Three treatments were considered: flask exposed to light (“light flask”), flask shaded from light (“dark flask”), and flask exposed to light but with sterile leaves (“sterile flask”). A control without deuterated PAH was also set up. Flasks were kept on a laboratory bench at room temperature for 50 days. One leaf was sampled from each flask at times of 15 and 50 days and analyzed for deuterated OH-PAH and di-OH-PAH determination.

### Plant Species, Leaf Sampling, and Washing Procedures

Holm oak leaves collected in a small village located in a semiurban area characterized by exposure to PAH and PM sources (Veniano, CO, Italy) were used to performed microcosm experiments. Holm oak was chosen being an evergreen plant species that accumulates PAH and PM from air^25^ and recently used to investigate pyrene mineralization by phyllosphere microorganisms.^16^ Leaves of approximately the same size (surface area of 10 cm^2^, fresh weight of 0.300 g) were collected and stored in a sterile plastic bag at +4 °C until the start of the experiment (within 3 h). Leaves to be used as the sterile control in WCS experiments were sterilized following a modified version of the procedure reported in Siriratruengsuk et al.^19^ Briefly, phyllosphere microorganisms were removed from the collected leaves by immersing 30 freshly collected leaves in 100 mL of 0.1 M potassium phosphate buffer (pH 7.0) and shaking them at 200 rpm for 15 min. Samples were later sonicated in an ultrasonic bath for 60 min to release the adhering microorganisms. Leaves used as the sterile control in the ACS experiment were sterilized with a 3% hydrogen peroxide solution (1 min wash) similarly to Saldierna Guzmán et al.^26^ to reduce the contact time of leaves with the aqueous solution

### Microorganism Inoculum, Medium, and Cultivation

Autochthonous phyllosphere microorganisms were obtained by washing holm oak leaves as described in the Plant Species, Leaf Sampling, and Washing Procedures section. The washing solution was centrifuged at 3000 rpm for 15 min, and the obtained pellet was used as an inoculum for the WCS-PhMi experiment. More specifically, an inoculum of 10^4^ CFU (colony forming units)/mL was used for the WCS-PhMi experiment to reproduce a realistic cell density as those found in the phyllosphere of different plant species (10^5^–10^6^ CFU/g of fresh leaves).^19^ For the WCS-EL experiment, the phyllosphere microbial communities already present on leaves were used. M9 mineral medium (Na_2_HPO_4_ 6 g/L, KH_2_PO_4_ 3 g/L, NH_4_Cl 1 g/L, NaCl 0.5 g/L, MgSO_4_ 0.12 g/L, CaCl_2_ 0.01 g/L) was employed to guarantee the minimal salt base formulation for the microbial growth in WCS-PhMi and WCS-EL experiments.

### Sample Analysis

Holm oak leaves were initially analyzed to determine native PAH concentrations, native PAH hydroxy- and dihydroxy metabolite concentrations, PM number and amount, bacterial and fungal plate counts, and bacterial community structure at the beginning of the experiment. During the experiment, deuterated PAH metabolites were measured in the WCS-PhMi system mineral medium and in leaves of WCS-EL and ACS-EL samples. The bacterial community structure was analyzed at the end of the experiment in the WCS systems. More details are reported in the Supporting Information. Briefly, samples were extracted and analyzed with gas chromatography–mass spectrometry (GC-MS) for PAH determination and with liquid chromatography–high-resolution mass spectrometry (LC-HRMS Orbitrap) for PAH metabolite measurement. Target and suspect screening analyses were performed for PAH metabolites obtaining a confidence identification level “1, confirmed by standards” for 9-hydroxy-phenanthrene (or 9-phenanthrol) and 1-hydroxy-pyrene (available standards) and level “3, tentative candidates” for the other compounds (for which standards were not available) according to Schymanski et al.^27^ Scanning electron microscopy (SEM) was used for PM analyses. The spread plate method was used to quantify cultivable phyllosphere bacteria and fungi, while the 16rRNA gene metabarcoding analyses were employed to study the structure of the phyllosphere bacterial community.

### Data Elaboration

The conversion ratio of parent compounds into metabolites (CR) was calculated for each microcosm system and treatment as well as field samples as follows:

$$CR\left(\%\right)=\frac{M}{PC}\cdot 100$$

where CR (%) is the conversion ratio, M is the highest amount (ng) of metabolite produced during the experiment or measured at T0 in holm oak leaves (sum of all labeled or native isomers of the same metabolite, respectively), and PC is the amount (ng) of the specific parent compound measured at the beginning of the experiment (labeled or native). Please note that for laboratory experiments CR refers to deuterated PAHs, while for field samples it refers to native PAHs.

## 3 Results and Discussion

### Determination of Native PAH Concentrations in Holm Oak Leaves

Total PAH concentration was 200 ng/g of dw. Phenanthrene was the most abundant compound (55%), followed by pyrene (19%) and fluoranthene (12%) (Table 2 and Figure S1). These data were in line with those reported in the literature for other plant species located in urban and semiurban areas in Italy. For example, Franzetti et al.^13^ measured PAHs in leaves of magnolia (*Magnolia grandiflora*) and cedar (*Cedrus deodara*) collected in an urban park in Milan in 2016, showing concentrations ranging from about 100 ng/g dw in fall to about 500 ng/g dw in winter. Terzaghi et al.^28^ detected up to 170 ng/g dw of total PAH in leaves of cornel (*Cornus mas*) and maple (*Acer pseudoplatanus*) collected in a semiurban area located in Como in spring 2007.

**Table 2:** Native PAH, OH-PAH, and di-OH-PAH Concentrations in Holm Oak Leaves at T0^a^

PAH and PAH metabolites	Leaf Conc. (ng/g dw)
PHE	110.4
FLUOT	26.0
PYR	37.5
B[a]ANTH	3.3
CHR	16.2
B[b]FLUOT	6.9
Sum PAH	200.3
OH-NAP	2.4
ACY-OH	0.1
ACE-OH	6.3
FLUO-OH	4.5
PHE-OH/ANTH-OH	3.1
FLUOT-OH/PYR-OH	1.0
B-[a]ANTH-OH/CHR-OH	0.4
Sum OH-PAH	17.9
di-ACY-OH	0.1
di-ACE-OH	24.2
di-FLUO-OH	11.4
di-PHE-OH/ANTH–OH	1.5
Sum di-OH-PAH	37.2

a NAPH, ACY, ACE, FLUO, ANTH, B­[a]­PYR, PER, DB­[a,h]­ANTH, I­[cd]­PYR, B­[g,h,i]­PER, B­[b]­FLUOT-OH/B­[a]­PYR-OH/PER-OH, DB­[ah]­ANTH-OH, B­[ghi)­PER-OH/I­[cd]­PYR-OH, COR-OH, di-OH-NAP, di-FLUOT-OH/di-PYR-OH, di-B-[a]­ANTH-OH/di-CHR-OH, di-B­[b]­FLUOT-OH/di-B­[a]­PYR-OH/di-PER-OH, di-DB­[ah]­ANTH-OH, di-B­[ghi)­PER-OH/di-I­[cd]­PYR-OH, di-COR-OH were < LOQ.

### Determination of Native PAH Metabolite Concentrations in Holm Oak Leaves

Several OH-PAH and di-OH-PAH were found using target and suspect screening analyses (Table 2 and Figure S1). More specifically, total OH- and di-OH-PAH concentrations were about 25% of the total PAHs. The most abundant compounds were OH-ACE, OH-FLUO, OH-PHE/OH-ANTH, di-OH-ACY, and di-OH-FLUO. PAH metabolites represent an important amount of total PAH and can derive from air or PM deposition to the leaf surface (i.e., from primary sources), or they could be produced on leaf surfaces by PAH photo- and/or biodegradation. These PAH metabolites were never detected in plant leaves collected in the field. However, some studies are available about their presence in air PM.^29–33^ In general, only a few compounds (i.e., OH-NAP, OH-FLUO, OH-PHE, and OH-PYR) were measured, since different analytical techniques (such as GC-MS and HPLC-fluorescence detection) were used for target analyses of the compounds for which analytical standards were available. High resolution mass spectrometry analyses performed in the current work allowed suspect screening analysis of additional metabolites. Avagyan et al.^34^ performed target analysis and suspect screening of OH-PAHs in air PM using HPLC-HRMS (Orbitrap) and identified additional PAH metabolites including OH-NAP, OH-PHE, OH-PYR, OH-CHR, OH-B­[a]­PYR (target analysis) and OH-ACY, OH-FLUO, OH-FLUOT, OH-B­[ghi]­FLUOT, OH-B­[a]­PYR (suspect screening). Some evidence of nitro-PAHs occurring in plant leaves is also available in the literature, but their presence is ascribed mainly to air–leaf transfer rather than degradation reactions on leaf surfaces.^35^

### Measurement of Particulate Matter Number and Amount on Holm Oak Leaves

The results obtained from PM counts are presented in Table S4 and Figure S2. Total PM (represented by PM10) was about 55 μg/cm^2^. These data are consistent with those presented in the literature for *Quercus ilex* and *Quercus pubescens* leaves.^36–38^ Data show the clear predominance of small particles (0.1–1 μm) in terms of the number of particles and the absence of particles larger than 10 μm in diameter (Figure S3).

### Microbial Count and Determination of Bacterial Community Structure of Holm Oak Leaves

The cultivable microbial count, both bacterial and fungal, showed around 10^6^ CFU/g or 10^4^ CFU/cm^2^ of fresh leaves (Table S5), similarly to other studies.^19^ It is however well-known that only a small fraction of the total microbial community is cultivable (0.1–8.4%).^39^ Based on the 16S rRNA gene sequencing analysis (GenBank accession numbers PV835621 and PV836504), the alpha-diversity of the bacterial community, in holm oak leaves at T0, shows the highest Chao1 index, (denoting species richness), corresponding to 392 compared to the microbial diversity detected at the end of the experiments (Figure S10). The Shannon and Simpson diversity indices were, respectively, 4.33 and 0.97.

The phyllosphere bacterial community structure analysis showed that the most abundant phyla were Proteobacteria (40%) and alpha Proteobacteria (33%) together with gamma Proteobacteria (7%), Bacteriodota 40%, Actinobacteriota 14%, and Deinococcus-Thermus 3% (Figure S4). These phyla were recently reported as predominant in bacterial phyllosphere.^40,41^ At the genus level, the relative abundance data evidence the presence of specific epiphytic and endophytic bacterial genera, like *Hymenobacter*, 1174-901-12, *Massilia*, *Deinococcus*, *Methylobacterium-Methylorubrum*, *Amnibacterium*, and *Curtobacterium*. These genera are associated to adaptation capabilities to stressed environmental conditions, such as temperature, light, UV radiation, low water content, nutrient availability, frost formation, and biosurfactant release facilitating sugar availability together with capabilities to enhance plant growth and yield, as well as being nitrogen fixers (Figure S4).^40,42^ It is also important to highlight the presence of bacterial genera such as *Variovorax*, *Sphingomonas*, *Pseudomonas*, *Pseudoxanthomonas*, *Sphingobium*, and *Novosphingobium*. These genera are known for being capable of degrading various organic compounds such as isoprene, methanol, xylene, PAHs, and organochlorine pesticides (OCPs) (Figure S4).^13,14,20,43^

### Metabolite Production in WCS-PhMi Microcosm

In this system, only two metabolites were found: OH-phenanthrene-d9 (OH-PHE-D9) and OH-chrysene-d11 (OH-CHR-D11). Mono-OH metabolites of naphthalene, acenapthene, pyrene, and perylene were not detected, as well as di-OH-metabolites of all parent compounds. Figure 2 and Figure S5 show the temporal variability of OH-PHE-D9 and OH-CHR-D11 metabolites for different treatments. In light treatment, metabolites appeared on day 7, generally with higher concentrations than dark and sterile ones; in dark treatment, metabolites appeared at day 7 for phenanthrene-d10 or at day 14 for chrysene-d12. In sterile treatment, metabolites of chrysene-d12 appeared on day 28, while phenantrene-d10 metabolite production was negligible. The lack of metabolites detected in the sterile treatment exposed to light conditions (concentrations < LOQ or lower than the light treatment) suggested that phyllosphere microorganisms have a role in producing OH-PAH metabolites in light and dark treatments. In fact, degradation was higher for phenanthrene-d10 and chrysene-d12 when light was present, and treatment was not sterile, possibly showing an interplay between light-based reactions and microbial degradation. The amount of metabolites produced represented up to 8% (for phenanthrene-d10) and to 0.9% (for chrysene-d12) of the initial amount of deuterated parent compound spiked in MM. No metabolites were detected in the treatment without PAH initial spike.

**Figure 2:**
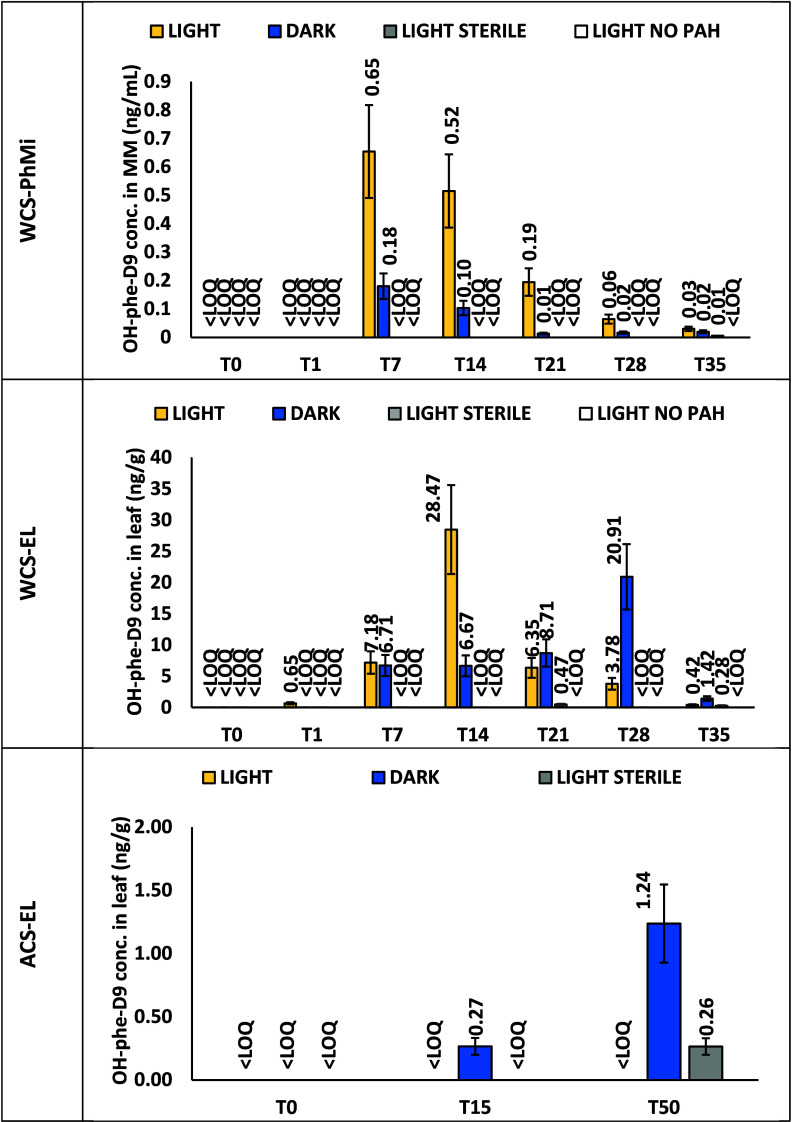
Deuterated OH-phenanthrene (OH-PHE-D9) concentrations produced in the three microcosms. Microcosm acronyms are as follows: WCS-PhMi stands for water contact system, phyllosphere microorganisms”, WCS-EL for “water contact system, entire leaf”, and ACS-EL for “air contact system, entire leaf”. Error bars represent the coefficient of variation (CV%) (analytical variability).

### Metabolite Production in WCS-EL Microcosm

In this system, only three metabolites were found: OH-phenanthrene-d9, OH-pyrene-d9 (OH-PYR-D9) and OH-chrysene-d11. Naphthalene, acenaphthene, and perylene mono-OH metabolites, as well as di-OH-metabolites of all parent compounds, were not detected. Figure 2 and Figure S5 show the temporal variability of OH-PHE-D9, OH-PYR-D9, and OH-CHR-D11 metabolites for the different treatments. In light treatment, metabolites appeared on day 1, but at very low concentrations (<1 ng/g). Concentrations reached maximum values on day 14 (29, 3, and 30 ng/g for OH-PHE-D9, OH-PYR-D9, and OH-CHR-D11 respectively). In dark treatment, metabolites appeared after 7 days for phenanthrene-d9 and chrysene-d12 and after 21 days for pyrene-d9, with concentrations comparable to those of the light treatments for the same time (7, 0.1, and 2 ng/g for OH-PHE-D9, OH-PYR-D9 and OH-CHR-D11 respectively). Concentrations reached maximum values on day 28 (21 and 0.3 for OH-PHE-D9 and OH-PYR-D9, respectively) and at day 35 for OH-CHR-D11 (21 ng/g). Metabolite production was negligible in sterile treatment. Again, concentrations obtained for sterile treatment exposed to the light condition (with concentrations generally lower than those of light and dark treatments) suggested again the interplay role of phyllosphere microorganisms and light conditions in enhancing degradation. This is similar to the previous experiment, where light only mediated reactions in absence of a microbial community, inducing negligible metabolite production. The amount of metabolites produced represented up to 1.4% (for phenanthrene-d10 and chrysene-d12) and to 0.3% (for pyrene-d10) of the initial amount of deuterated parent compound spiked on holm oak leaves. No metabolites were detected in the treatment without PAH initial spike.

### Metabolite Production in ACS-EL Microcosm

In this system, only one metabolite was found: OH-phenanthrene-d9. Naphthalene, acenaphthene, and perylene OH-metabolites were not detected, as well as di-OH-metabolites (pyrene was not spiked at T0). Some chrysene metabolites were also identified, but their concentrations were < LOQ. OH-PHE-D9 appeared after 15 days in dark treatment (0.27 ng/g) and reached a maximum concentration after 50 days (1.24 ng/g). In light treatment, no metabolites were found, while in the sterile one, they appeared after 50 days. Figure 2 shows the temporal variability of the OH-PHE-D9 metabolite for the different treatments. The amount of metabolites produced represented less than 1% of the initial amount of deuterated parent compound spiked on leaves.

### Comparison of OH-PAH Metabolite Production in Lab and Field

The three experiments showed different results in terms of the number of metabolites and isomers for each metabolite produced and the parent compound to metabolite conversion ratio. WCS-PhMi mesocosm showed the production of just one isomer of OH-PHE-D9 and OH-CHR-D10, but the highest conversion ratio (Figure 3), especially for phenanthrene-d10.

**Figure 3:**
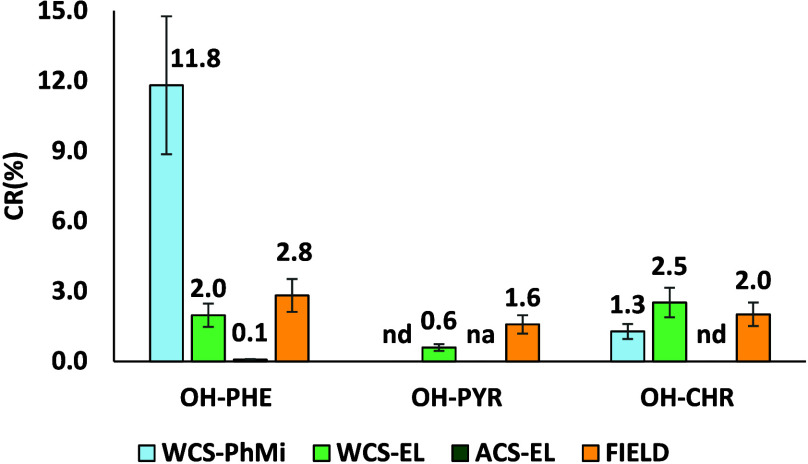
Parent compound to metabolite conversion ratio for the three experimental systems and field samples. Please note that for laboratory experiments, CR refers to deuterated PAHs, while for field samples it refers to native PAHs. Microcosm acronyms are as follows: WCS-PhMi stands for “water contact system, phyllosphere microorganisms”, WCS-EL for “water contact system, entire leaf”, and ACS-EL for “air contact system, entire leaf”. Error bars represent coefficient of variation (CV%) (analytical variability). nd = not detected; na = not available.

This was probably due to the experimental conditions that were optimized to accelerate the degradation reactions (i.e., agitated mineral medium). The number of PAH metabolites and their isomers increased when entire leaves (WCS-EL and ACS-EL) were used, rather than the inoculated phyllosphere microorganisms only (WCS-PhMi) suggesting enhanced effects of the phyllosphere microbial community. An additional possible source of PAH metabolites in a real environment could be the direct transformation of the PAHs by the leaves through cytochrome P-450 enzymes.^44,45^ This effect was not evident in this study, since no degradation (including therefore plant enzymatic activities) appeared in the sterilized controls. This could also be due to the sterilization treatment, which might have inactivated plant biochemical response.

Three isomers appeared for OH-PHE-D9 and OH-CHR-D10 in WCS-EL and ACS-EL, similar to field samples (Figures S6 and S8), while just one was for OH-PYR-D9 (Figure S7). The number of native OH-PAH isomers identified in holm oak leaves collected in field at the beginning of the experiment corresponded to that of labeled OH-PAH isomers. Their chemical identities were verified acquiring the exact masses of the corresponding unlabeled metabolites and evaluating the retention times, which were similar. However, their identity should be confirmed with analytical standards. In terms of parent compound to metabolite conversion ratio, the WCS-EL system showed results comparable to those of holm oak leaves at T0. This could suggest that OH-PAH detected in field samples could be produced by photo- and biodegradation of native PAHs occurring on leaf surface. On the contrary, CR was lower for the most realistic system (ACS-EL). Probably, microbial growth was slower in this microcosm, although wet sand was used as support to guarantee leaf humidity and/or deuterated-PAH volatilization from leaves to air was faster than from mineral medium.

For naphthalene-d8 and acenaphthene-d10, no OH and di-OH PAH metabolites were detected in experimental systems, probably due to the high volatility of the parent compounds and the possibly reduced concentrations during the experiment; their native metabolites were instead detected in field samples. Perylene-d12, the most hydrophobic compound, was not converted to OH and di-OH metabolites, and this result was in line with field data for native compounds. While in the literature several studies reported the production of naphthalene and acenaphthene OH and di-OH metabolites by several microorganisms,^46–49^ no data were found about perylene biodegradation and photodegradation.

### Phyllosphere Bacterial Community in WCS Systems

The different results obtained with the two WCS systems are also supported by microbiological analyses. Alpha-diversity (i.e., Choi1 index)^50^ was much higher in the WCS-EL system, both in light and dark conditions, and more similar to that measured in holm oak leaves at T0, suggesting a higher bacterial richness in this leaf system (Table S7 and Figure S10). Moreover, beta-diversity analysis showed that the bacterial community structure of the WCS-EL system (both light and dark conditions) was close to that of holm oak leaves at T0, confirming the similar parent compound to metabolite conversion ratio (Figure S11). In the WCS-EL exposed to light, bacterial genera involved in organic pollutant and PAH degradation processes were observed (Figure S12). These genera were, e.g., *Devosia* 23%, *Sphingobium* 9%, *Novosphingobium* 4%, *Sphingomonas* 7%, *Variovorax* 8%, *Pseudomonas* 2%, *Pantoea* 2%. The high relative abundance of the genus Devosia in WCS-EL (light treatment) is particularly interesting, because Devosia was reported having an important role in pyrene degradation.^51^ In WCS-EL dark conditions, bacterial genera involved in both organic pollutants and PAH degradation processes were also present but less abundant. Furthermore, a decrease of pigmented bacterial genera associated with photoprotection mechanisms was also noticed. In WCS-EL, sterile conditions, a high relative abundance of the endophytic bacterial genera was observed (like *Allorhizobium–Pararhizobium–Rhizobium* 24% and *Delftia* 15%), probably indicating that the adopted sterilization procedure mainly removed the epiphytic bacteria. Bacterial community structures in WCS-PhMi systems showed lower alpha-diversity indices (Table S7 and Figure S10) and different beta-diversity (Figure S11), compared to those of WCS-EL systems and holm oak leaves at T0. A lower abundance of some bacterial genera associated with organic pollutants degradation processes, like *Devosia*, *Pseudomonas*, *Sphingobium*, *Novosphingobium*, was also noticed (Figure S12).

### Environmental Significance of These New Aspects of FFE

In this work, native OH-PAH and di-OH-PAH metabolites were measured for the first time in leaf samples collected in field. Their production was investigated through different experiments of increasing complexity. The results highlight the importance of phyllosphere microorganisms and light in the degradation of parent PAH and in the formation of OH-PAH. These degradation reactions could help to maintain a high air to leaf PAH gradient since these compounds could be continuously removed. This could be particularly important for FFE, making the biological pump even more efficient since it involves not only PAH leaf uptake but also phyllosphere removal of the same chemicals. Additionally, the metabolites produced, being more polar and less hydrophobic, may likely be washed off during rainfall events and be transported to the terrestrial environment, restoring the PAH gradient. Although the experiments were designed as a first step, under simplified conditions, to investigate the production of OH-PAHs metabolites in the phyllosphere, they clearly demonstrated that the production of metabolites occurs in the leaf environment. In these experiment conditions, the amounts of metabolites produced are generally low. However, in a natural environment, with higher light intensity, higher humidity conditions (due to the water release during evapotranspiration), and possibly a larger bacterial diversity, metabolite production could be more important. Since a microcosm does not fully reproduce the exposure conditions of leaves in a natural system, it helped in understanding the main factors affecting the degradation processes and could be important in planning more realistic experiments, for example, using whole plants instead of single, detached, leaves. However, further studies are necessary to better define the source and the processes involved in PAH metabolites formation, in order to quantify the contribution of phyllosphere biodegradation on PAH removal from air and its precise role in forest filter effect process.

### Limitations of the Study

This work was aimed at assessing whether the phyllosphere could have a role in degrading PAHs, in connection with other factors. One of these factors was light, which was relatively weak compared to outdoor sunlight and may not fully mimic natural photodegradation.

In the WCS/ACS-EL experiment, chemicals were added dissolved in acetone: acetone could have altered the structure of the cuticular wax, with the effect of potentially distributing PAHs through the depth of the epicuticular wax layer. This might have had the effect of depositing PAHs at a certain distance from the surface layer, where microbes would be present. This means that the degradation observed could have been lower than expected.

Also, future works should be dedicated to highlight the role of specific epi- and endophytes in the biodegradation processes and the role of plant enzymes in a sterilized system. The experiments should also be carried out by evaluating additional conditions. For example, optimizing the microcosms with sterile controls shaded from light will help to understand the abiotic degradation.

## Supplementary Material



## Data Availability

Sequence data are openly available at GenBank (https://www.ncbi.nlm.nih.gov/genbank/), accession numbers from PV835621 to PV836504.

## Notes

Additional information about experimental design, sample analyses, and experiment results (PDF)
