# Supplementary material for: Forest Filter Effect Revisited: First Evidence That Polycyclic Aromatic Hydrocarbon Metabolites Are Produced on Leaves by Biodegradation and Photodegradation
Source: Environ Sci Technol. 2025 Sep 15;59(38):20738–47. doi: 10.1021/acs.est.5c09252 (PMC12489981; doi:10.1021/acs.est.5c09252)
Supplement: Supplementary file 1 [file es5c09252_si_001.pdf]

SUPPORTING INFORMATION

Forest Filter Effect Revisited: First Evidence That Polycyclic Aromatic Hydrocarbon Metabolites Are Produced on Leaves by Bio- and Photo-Degradation

Elisa Terzaghi[a*], Corinne Bertipaglia[a], Elisabetta Zanardini[a], Davide Siniscalchi[a], Renzo Bagnati[d], Alice Passoni[d], Laura Rampazzi[b], Cristina Corti[b], Josè-Julio Ortega-Calvo[c], Rosa Posada-Baquero[c], Antonio Di Guardo[a]

[a] Department of Science and High Technology, University of Insubria, Via Valleggio 11, 22100 Como, Italy
[b] Department of Human Sciences and Innovation for the Territory, University of Insubria, Via Sant'Abbondio 12, 22100 Como, Italy
[c] Instituto de Recursos Naturales y Agrobiologiá de Sevilla (IRNAS-CSIC), Avda. Reina Mercedes, 10, E-41080 Seville, Spain
[d] Department of Environmental Health Sciences, Istituto di Ricerche Farmacologiche "Mario Negri" IRCCS, Via Mario Negri 2, 20156 Milan, Italy

22 pages, 12 figures, and 7 tables

TABLE OF CONTENTS

S1 SAMPLE ANALYSES 2
S1.1 Chemical and reagents 2
S1.2 Chemical analyses 3
S1.2.1 Native PAH and native PAH metabolites concentration in leaves 3
S1.2.2 Deuterated PAH metabolites concentrations in leaves and mineral medium 4
S1.2.3 Quality assurance/quality control (QA/QC) 7
S1.3 Particulate matter analyses on leaves 8
S1.4 Microbiological analyses 9
S1.4.1 Cultivable bacterial and fungal counts 9
S1.4.2 Bacterial community structure 9
S2 RESULTS 11
S2.1 Native PAH and PAH metabolites in holm oak leaves at T0 11
S2.2 PM number and amount on holm oak leaves at T0 12
S2.3 Cultivable bacterial and fungal counts and bacterial community analysis of holm oak leaves at T0 13
S2.4 Pyrene and chrysene deuterated metabolites produced in mesocosm experiments 15
S2.5 Metabolite isomers produced in mesocosm experiments and in field 16
S2.6 Bacterial community structure analysis in microcosm experiments 18
REFERENCES 22

* elisa.terzaghi@uninsubria.it

S1 SAMPLE ANALYSES

S1.1 Chemical and reagents

Acenaphthene (ACE), acenaphthylene (ACY) fluorene (FLUO), phenanthrene (PHE), anthracene (ANTH), fluoranthene (FLUOT), pyrene (PYR), benz[a]anthracene (B[a]ANTH), chrysene (CHR), benzo[b]fluoranthene (B[b]FLUOT), benzo[a]pyrene (B[a]PYR), benzo[g,h,i]perylene (B[ghi]PER), indeno[c,d]pyrene (I[cd]PYR), were purchased from Supelco (QTM PAH mix, 2000 ng/µL in methylene chloride). Perylene (PER) was purchased from Sigma-Aldrich. Deuterated standards (Naphthalene-d8, Acenaphthene-d10, Phenanthrene-d10, Chrysene-d12, Perylene-d12) were purchased from Supelco (Semivolatile Internal Standard mix, 2000 ng/ µL in methylene chloride). Pyrene-d10 was purchased from Sigma Aldrich. PAH hydroxy metabolites (9-phenanthrol and 1-hydroxypyrene) were purchased from Merck. Solvents used were acetone (pesticide grade, Honeywell), cyclohexane (pesticide grade, Honeywell), ethylacetate (pesticide grade, Honeywell) and acetonitrile (LC-MS grade, Honeywell), methanol (pesticide grade, Sigma-Aldrich), water (in house Milli-Q apparatus). Ammonium acetate was obtained from Honeywell. PCB 136 was purchased by Accustandard. Supelclean ENVI-18 SPE cartridges (3 mL) were purchased from Merck. Sand (particle size comprised between 300 to 350 µm) was purchased from Zolux (France). Anhydrous sodium sulfate was purchased from Sigma-Aldrich; silica gel 60 for column chromatography (0.063-0.200 mm) was purchased from Merck; neutral aluminum oxide was purchased from Sigma-Aldrich. M9 mineral medium salt were obtained from several suppliers: Na_2HPO_4 (>99%, Chemlab); KH_2PO_4 (>99.5%, Honeywell Fluka); NH_4Cl (99.5%, ITW Reagents); NaCl (99.5%, ITW Reagents); $MgSO_4$ (99.5%, ITW Reagents); $CaCl_2$ (>93%, Honeywell Fluka). Tryptic Soy Agar (TSA) and Sabouraud Glucose Agar (SGA) were purchased from Becton Dickinson GmbH. Sterile physiological solution was purchased from Liofilchem.

S1.2 Chemical analyses

S1.2.1 Native PAH and native PAH metabolites concentration in leaves

To determine the initial concentrations of native PAHs in holm oak leaves, approximately 10 g of fresh leaves were blended and mixed with anhydrous sodium sulfate (Na_2SO_4) and then spiked with 50 µL of a recovery standard mixture (deuterated PAH-mix, 4 ng/µL). Samples were extracted by sonication (Branson mod. 8510) with 100 mL of a mixture of cyclohexane/ethylacetate 1/1 for 1 h at 25 °C and then filtered on a Na_2SO_4 column to remove the leaf biomass. Leaf extracts were first cleaned-up with gel permeation chromatography (GPC) (US EPA, 1994), and then an additional silica-alumina cleanup step was performed with columns packed with sodium sulphate and with silica and alumina oxide similarly to (US EPA, 1996). After the clean-up processes the samples were concentrated up to 2 mL and 10 µL of internal standard (PCB 136, 20 ng/µL) were added to each sample. Samples were then concentrated under N_2 flow to 100 µL and injected in a GC-MS system (Agilent 7890 GC coupled to an Agilent 5977 mass selective detector). The chromatographic separation was achieved on a HT8 column (50 m, i.d. 0.22 mm, film thickness 0.25 µm, SGE). The oven temperature program was 70°C x 0 min, to 155 °C x 0 min at 5 °C/min, to 335 °C x 23 min at 6.5 °C/min. Separation of PAHs was achieved in 68 min. The injector and detector temperatures were at 280°C and 340°C respectively. The injection volume was 2 µL. The carrier gas used was helium, at a flow rate of 0.75 mL/min. PAH were analyzed in selected ion monitoring mode (SIM). The monitored masses and retention times are reported in **Table S1.** 16 PAH were quantified. Internal standard calibration method was used for the quantification. Leaf extract used for native PAH determination was injected in a UHPLC-HRMS system after solvent exchange (cyclohexane/ethylacetate 1/1 was substituted by acetonitrile) for native PAH metabolite detection.

Table S1 PAH monitored masses and retention times (RT)

PAH	RT (min)	Ion 1	Ion 2	Ion 3
**Naphthalene**	17.24	128	127	129
**Acenaphthylene**	24.70	152	151	76
**Acenaphthene**	25.43	154	153	152
**Fluorene**	25.53	166	165	167
**Phenanthrene**	31.75	178	176	179
**Anthracene**	32.07	178	179	176
**Fluoranthene**	36.77	202	200	101
**Pyrene**	37.74	202	200	101
**Benz[a]anthracene**	42.43	228	226	229
**Chrysene**	42.60	228	226	229
**Benzo[b]fluoranthene**	47.06	252	250	253
**Benzo[a]pyrene**	48.76	252	253	250
**Perylene**	49.01	252	250	125
**Benzo[g,h,i]perylene**	54.68	276	274	138
**Dibenzo[a,h]anthracene**	55.25	278	276	139
**Indeno[1,2,3-cd]**	57.27	276	274	138
**PCB 136**	37.34	360	362	364
**Naphthalene d8**	11.57	136	137	134
**Acenaphthene d10**	25.18	164	162	160
**Phenanthrene d10**	31.67	188	189	184
**Chrysene d12**	42.60	240	236	241
**Perylene d12**	48.925	264	263	265

S1.2.2 Deuterated PAH metabolites concentrations in leaves and mineral medium

Deuterated PAH metabolites were extracted from leaf samples (i.e., one leaf at a time) using 40 mL of cyclohexane/ethylacetate 1/1 for 1 h at 25°C using a sonicator. The same procedure was repeated using 40 mL of acetone. The extract was purified with silica and alumina oxide, using 10 mL of the cyclohexane/ethylacetate 1/1, 10 ml of acetone and 10 ml of acetonitrile as eluting solvents. After sample volume reduction to 1 mL, the extract was centrifuged for 15 min at 12000 rpm and 5°C. Supernatant was collected, concentrated to dryness under N_2 flow and finally reconstituted in 1 mL of acetonitrile, and transferred into a 1 mL injection amber glass vial. Deuterated OH-PAHs and di-OH-PAHs in mineral medium were extracted according to (Marques et al., 2021). Briefly, the SPE C18 cartridges were conditioned with 5 ml of methanol followed by 10 mL of ultrapure water. 10 mL of mineral medium were loaded on SPE cartridges and purified with 10 mL of methanol/water (40/60); the compounds were eluted with 7 mL of acetonitrile and evaporated to 100 µL under N_2

flow. Analyses of the leaf and mineral medium samples were performed using a UHPLC Thermo Fisher Vanquish coupled to a Thermo Fisher Orbitrap Exploris 120 high resolution mass spectrometer operating in ESI negative ion full scan MS (200−800 u) at 60,000 resolution and in data dependent MS^2 at 30,000 resolution and 30−60 NCE collision energies. The ionization conditions were optimized for acidic compounds and resulted in the formation of negative molecular ions $[M-H]^-$ for OH-PAH and di-OH-PAHs. With these instrumental conditions unmodified PAH were not ionized and consequently not detectable. The chromatographic separation was performed with Accucore C18 column, 50 × 2.1 mm ID, 2.6 µm phase (Thermo Fisher) using a gradient of ammonium acetate, 10 mM in water (A) and acetonitrile (B) at a flow rate of 350 µL/min. The chromatographic gradient was as follows: initially solvent A was set at 99% while B was set at 1%; B was then linearly increased to 99% in 19 min and kept constant for 10 min; then B was brought to 1% in 1 min, while A was set at 99% and kept constant for 6 min to equilibrate the column (total run time 36 min). Chromatograms obtained from the UHPLC-HRMS analysis were analyzed with the Freestyle software (ThermoFisher Scientific) for target and suspect screening analysis of PAH metabolites, based on their exact masses. Semiquantitative analyses of OH-PAHs were performed by extracting high resolution ion chromatograms (XIC) from full scan data, with a tolerance range of ±5 ppm. **Table S2** and **Table S3** reported the exact masses of the deprotonated form of each native and deuterated metabolite compound respectively. Reference standards of OH-phenanthrene (9-phenanthrol) and OH-pyrene (1-hydroxy-pyrene) were used to construct external calibration curves. Calculated concentrations were extrapolated by integrating the sum of all the chromatographic peaks of equally hydroxylated PAH metabolites, assuming they had an instrumental response equal to the compounds used as reference standard in external curve (9-phenanthrol was used to quantify all PAH metabolites with exception for pyrene metabolites). For this reason, the obtained data must be considered as an approximation of an accurate quantitation.

Table S2 Native PAH metabolite monitored masses and retention times (RT)

Substances	Molecular formula	RT (min)	Exact mass	$[M-H]^-$
hydroxy-naphthalene	$C_{10}H_8O$	9.89-10.25	144.05751	143.05024
di-hydroxy-naphthalene	$C_{10}H_8O_2$	n.f.	160.05243	159.04515
hydroxy-acenaphthylene	$C_{12}H_8O$	10.84-11.69	168.05751	167.05024
di-hydroxy-acenaphthylene	$C_{12}H_8O_2$	10.84-11.88	184.05243	183.04515
hydroxy-acenaphthene	$C_{12}H_{10}O$	11.38	170.07316	169.06589
di-hydroxy-acenaphthene	$C_{12}H_{10}O_2$	11.37-11.61	186.06808	185.06080
hydroxy-fluorene	$C_{13}H_{10}O$	11.56-12.10	182.07316	181.06589
di-hydroxy-fluorene	$C_{13}H_{10}O_2$	12.00-13.12	198.06808	197.06080
hydroxy-phenanthrene	$C_{14}H_{10}O$	12.10-12.93	194.07316	193.06589
di-hydroxy-phenanthrene	$C_{14}H_{10}O_2$	10.64-13.46	210.06808	209.06080
hydroxy-anthracene	$C_{14}H_{10}O$	12.10-12.93	194.07316	193.06589
di-hydroxy-anthracene	$C_{14}H_{10}O_2$	10.64-13.46	210.06808	209.06080
hydroxy-fluoranthene	$C_{16}H_{10}O$	13.23	218.07316	217.06589
di-hydroxy-fluoranthene	$C_{16}H_{10}O_2$	n.f.	234.06808	233.06080
hydroxy-pyrene	$C_{16}H_{10}O$	13.23	218.07316	217.06589
di-hydroxy-pyrene	$C_{16}H_{10}O_2$	n.f.	234.06808	233.06080
hydroxy-banzo[a]anthracene	$C_{18}H_{12}O$	13.86-14.72	244.08881	243.08154
di-hydroxy-banzo[a]anthracene	$C_{18}H_{12}O_2$	n.f.	260.08373	259.07645
hydroxy-chrysene	$C_{18}H_{12}O$	13.86-14.72	244.08881	243.08154
di-hydroxychrysene	$C_{18}H_{12}O_2$	n.f.	260.08373	259.07645
hydroxy-benzo[b]fluoranthene	$C_{20}H_{12}O$	n.f.	268.08881	267.08154
di-hydroxy-benzo[b]fluoranthene	$C_{20}H_{12}O_2$	n.f.	284.08373	283.07645
hydroxy-benzo[a]pyrene	$C_{20}H_{12}O$	n.f.	268.08881	267.08154
di-hydroxy-benzo[a]pyrene	$C_{20}H_{12}O_2$	n.f.	284.08373	283.07645
hydroxy-perylene	$C_{20}H_{12}O$	n.f.	268.08881	267.08154
di-hydroxy-perylene	$C_{20}H_{12}O_2$	n.f.	284.08373	283.07645
hydroxy-benzo[ghi]perylene	$C_{22}H_{12}O$	n.f.	292.08881	291.08154
di-hydroxy-benzo[ghi]perylene	$C_{22}H_{12}O_2$	n.f.	308.08373	307.07645
hydroxy-dibenzo[ah]anthracene	$C_{22}H_{14}O$	n.f.	294.10447	293.09719
di-hydroxy-dibenzo[ah]anthracene	$C_{22}H_{14}O_2$	n.f.	310.09938	309.09210
hydroxy-indeno[cd]pyrene	$C_{22}H_{12}O$	n.f.	292.08881	291.08154
di-hydroxy-indeno[cd]pyrene	$C_{22}H_{12}O_2$	n.f.	308.08373	307.07645
hydroxy-coronene	$C_{24}H_{12}O$	n.f.	316.08881	315.08154
di-hydroxy-coronene	$C_{24}H_{12}O_2$	n.f.	332.08373	331.07645

NOTES: n.f.=exact mass not found; when more than one peak appeared in the chromatogram a range of retention times was reported

Table S3 Deuterated PAH metabolite monitored masses and retention times (RT)

Substances	Molecular formula	RT (min)	Exact mass	$[M-H]^-$
hydroxy-naphthalene-d7	$C_{10}D_7HO$	n.f.	151.10145	150.09418
di-hydroxy-naphthalene-d6	$C_{10}D_6H_2O_2$	n.f.	166.09009	165.08281
hydroxy-acenapthene-d9	$C_{12}D_9HO$	n.f.	179.12966	178.12238
di-hydroxy-acenaphthylene-d6	$C_{12}D_6H_2O_2$	n.f.	190.09009	189.08281
hydroxy-phenanthrene-d9	$C_{14}D_9HO$	11.70-12.38	203.12966	202.12238
di-hydroxy-phenanthrene-d8	$C_{14}D_8H_2O_2$	n.f.	218.11829	217.11172
hydroxy-chrysene-d11	$C_{18}D_{11}H_1O$	13.49-14.07	255.15786	254.15058
di-hydroxy-chrysene-d10	$C_{18}D_{10}H_2O_2$	n.f.	270.14650	269.13922
hydroxy-pyrene-d9	$C_{16}D_9H_1O$	12.89	227.12966	226.12238
di-hydroxy-pyrene-d8	$C_{16}D_8H_2O_2$	n.f.	242.11829	241.11102
hydroxy-perylene-d11	$C_{20}D_{11}HO$	n.f.	279.15786	278.15058
di-hydroxy-perylene-d8	$C_{20}D_{10}H_2O_2$	n.f.	294.14650	293.13922

NOTES: n.f.=exact mass not found

S1.2.3 Quality assurance/quality control (QA/QC)

All analytical procedures were monitored using strict quality assurance and control measures. A laboratory blank (Na_2SO_4) was included at every sample analysis at a rate of 1 out of 4 leaf or water samples and extracted following the same procedures as for samples. At the beginning of each GC-MS injection sequence, and every two or three samples, vials containing 1 mL of cyclohexane were injected to clean up the syringe and the system to prevent cross contamination. Similarly, at the beginning of each LC-HRMS injection sequence and every two or three samples, vials containing 1 mL of acetonitrile were injected to clean up the system to prevent cross contamination. PAH calibration curves were determined for every injected series of samples. Limits of quantitation (LOQ) were calculated based on the lowest injected standards. LOQ for PAHs ranged between 0.14 to 14 ng/g dw for leaf samples depending on PAH compound. LOQ for PAH metabolites ranged between 0.09 to 0.24 ng/g fw for leaf samples and from 0.002 to 0.005 ng/mL for mineral medium samples. For PAH analyses in leaves recovery ranges from 51% to 94%. Analytical variability was obtained injecting 10 times the same standards and calculating the coefficient of variation (CV%). CV% was 15% for PAHs and 25% for OH-PAHs.

S1.3 Particulate matter analyses on leaves

The number of particles on leaf surface was counted using a Scanning Electron Microscope (SEM). SEM imaging was performed on one leaf, randomly chosen. One square portion of 1 cm^2 was cut near the center of the leaf, close to the main rib. The obtained sample was desiccated and observed on the top side without any further pre-treatment. A FEI/Philips XL30 ESEM scanning electron microscope was used, equipped with an Ametek Element EDX energy-dispersive X-ray spectrometer. Leaf portions were mounted on the sample holder by using double coated carbon conductive PELCO Tabs (Ted Pella, Inc.). SEM imaging was performed in low-vacuum mode (1 torr H_2O) using both the backscattered electron detector (BSE) with an incident electron energy of 20 kV and the gaseous secondary electron detector (GSE) with an incident electron energy of 10 kV. Five areas were selected for observation within the sample. The first one was centered at the center of the sample (point x=0, y=0). The whole sample was then divided into four quadrants. For each of them, an area centered at the center of the quadrant was observed. Each of these five areas was observed and photographed at different levels of magnification (GSE: 300x – BSE: 300-1000-2000-4000x). PM can be easily identified, especially in BSE images, appearing as bright particles on a darker leaf surface. Particles count was performed by using the open-source software ImageJ (v.1.54h), Fiji distribution (Schindelin et al., 2012): contrast and brightness were adjusted, then the images were smoothed and a B&W threshold ("default mode", "auto" settings) was applied to each image. Watershed function was then used in order to detect individual particles that compose aggregates of multiple particles. Finally, the particles were counted using the "analyze particles" function, also obtaining their size distribution calculated on their Feret diameters. Particle number was used, together with particle diameter, to calculate volumes (particle assumed to be spherical) and weight, assuming a density of 1.5 g cm^{-3} as in (Terzaghi et al., 2013).

S1.4 Microbiological analyses

S1.4.1 Cultivable bacterial and fungal counts

To determine the initial total number of phyllosphere microorganisms the spread plate method was used. Phyllosphere microorganisms were extracted from the holm oak leaves as described in the main text. The extracted solution was then centrifuged at 3000 rpm for 15 min to collect microorganism cells. The cell pellets were diluted in a sterilized 0.85% NaCl solution and plated on Tryptic soy agar (TSA) and Sabouraud Glucose Agar (SGA) to estimate the cultivable microbial count on leaf surface. The same procedure was used to obtain the inoculum for the WCS-PhMi experiment and to obtain the final cultivable microbial load count (at day 35) in WCS and WCS-PhMi experiments.

S1.4.2 Bacterial community structure

Bacterial community structure of holm oak phyllosphere was measured at T0 and at T35 for all the flasks. DNA was extracted from bacterial pellet using the "Blood & Tissue Genomic DNA Extraction kit" (Fisher Molecular Biology), according to manufacturers' protocol. The V3-V4 region of the 16S rRNA gene was amplified following the Illumina protocol "16S Metagenomic Sequencing Library Preparation" and utilizing the following primers:

- 16S-341f: 5'-TCGTCGGCAGCGTCAGATGTGTATAAGAGACAGCCTACGGGNGGCWGCAG-3';
- 16S-805r: 5'-GTCTCGTGGGCTCGGAGATGTGTATAAGAGACAGGACTACHVGGGTATCTAATCC-3'.

Quality control of the library was performed using the Fragment Analyzer (Agilent Technologies). Amplicon-seq libraries (metabarcoding) were generated using the Illumina 16S standard workflow by amplifying a portion of the hyper-variabile V3-V4 region of the 16S SSU rRNA gene using primers 341f and 805r. Sequencing was performed on the Illumina NextSeq platform (2x300 bp) and libraries demultiplexed by removing adapters. The libraries are directional (forward primer in R1 and reverse primer in R2). Sequences were imported and analyzed using QIIME2 (v2023.9) (Bolyen et al., 2019). First the primers were identified and removed using the 'cutadapt' plugin. Then, sequences were

quality-filtered, denoised (removal of artifacts and chimeras), and the amplicon sequence variants (ASV) were predicted using DADA2 (Callahan et al., 2016). The taxonomic assignment of the ASVs was performed using the 'naive Bayes' classification algorithm implemented in QIIME2, using the SILVA database (v138, https://www.arb-silva.de/documentation/release-138/). To enhance ASV classification performances the 515F/806R region of the 16S SSU rRNA gene was extracted from the reference database and used for ASVs classification. Statistical analyses were performed in R programming environment (R Core Team, 2023) using 'vegan' v2.6-4 package (Oksanen et al., 2022). The complete analysis metrics are reported in **Table S6** (Denoising and Amplicon Sequence Variant (ASVs) clustering statistics), **Figure S9** (Rarefaction curves), **Table S7** and **Figure S10** (Alpha-diversity indices) and **Figure S11** (Beta-diversity Principal Coordinates Analysis (PCoA). Krona chart (**Figure S4**) was generated using KronaTools.

S2 RESULTS

S2.1 Native PAH and PAH metabolites in holm oak leaves at T0

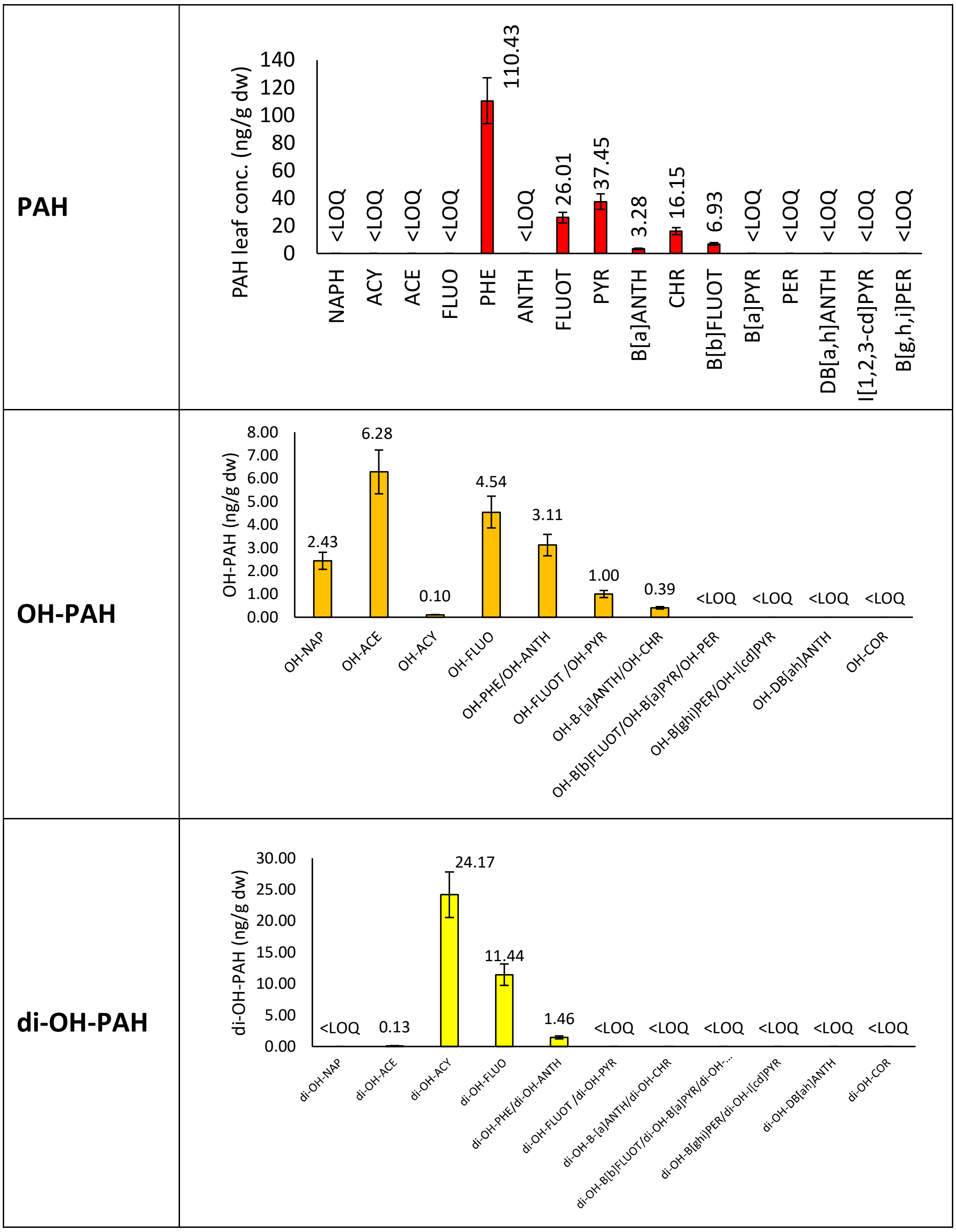

Figure S1 Native PAH, OH-PAH and di-OH-PAH concentrations in holm oak leaves at T0 (error bars (CV%) represent the analytical variability)

S2.2 PM number and amount on holm oak leaves at T0

Table S4 Particulate matter (number of particles and mass) on holm oak leaves at T0

	N/mm²			µg/cm²		
	AVERAGE	STDEV	CV%	AVERAGE	STDEV	CV%
**PM 0.1-1 µm**	146389	69791	48	1.91	0.91	48
**PM 1.1-2.5 µm**	11820	5766	49	5.41	2.64	49
**PM 2.6-10 µm**	2370	2897	122	46.55	56.88	122
**PM 10.1-100 µm**	0	0	0	0	0	0
**Total PM**	160579	72726	45	53.88	57.57	107

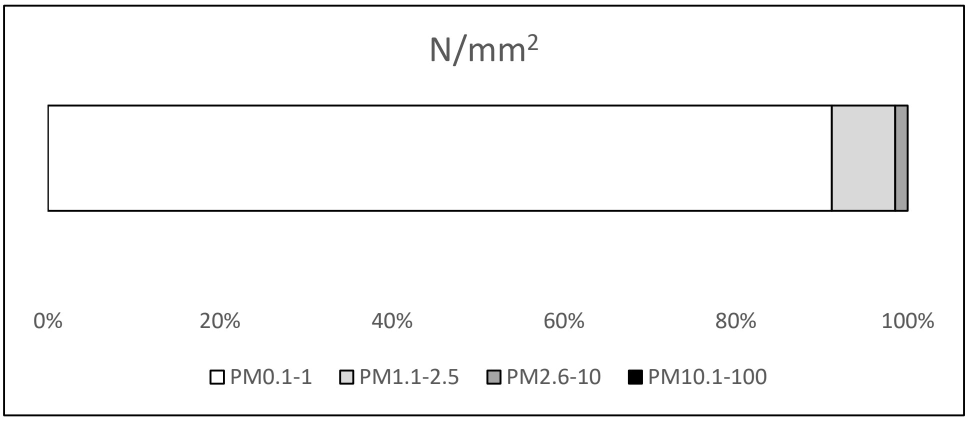

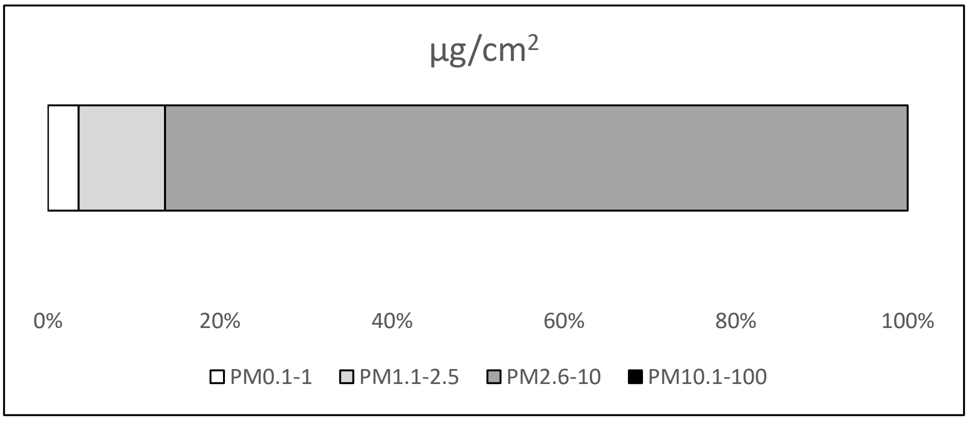

Figure S2 Particle size distribution on holm oak leaves at T0

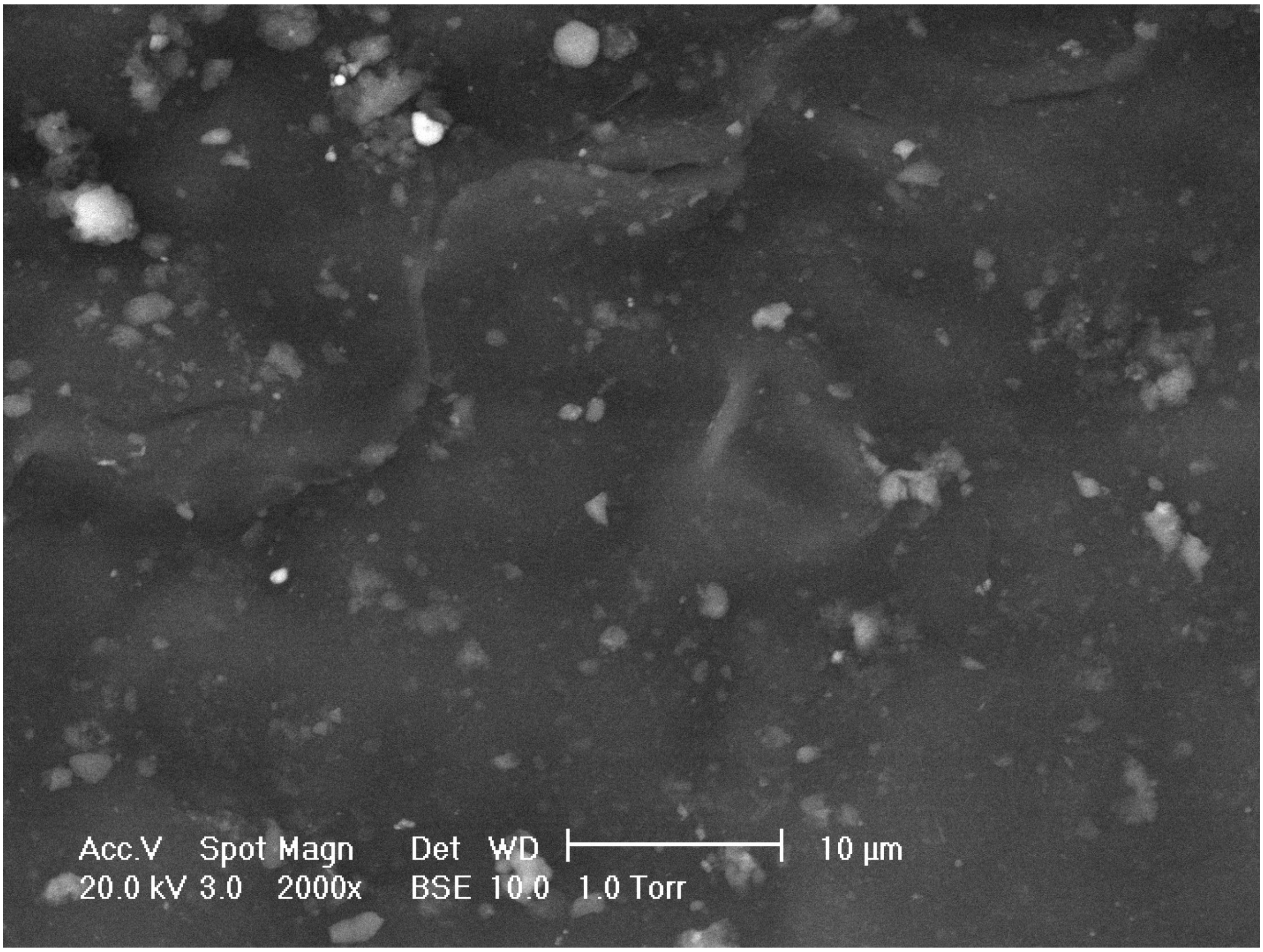

Figure S3 SEM image in Back Scattered Electrons mode, 2000X, of an holm aok leaf sample

S2.3 Cultivable bacterial and fungal counts and bacterial community analysis of holm oak leaves at T0

Table S5 Phyllosphere microbial load on holm oak leaves at T0

**Medium**	**Microorganisms**	**CFU/g**	**CFU/cm^2**
**TSA**	Bacteria	7.55E+05	2.35E+04
**SGA**	Fungi	1.52E+06	4.72E+04
	Bacteria + Fungi	2.28E+06	7.07E+04

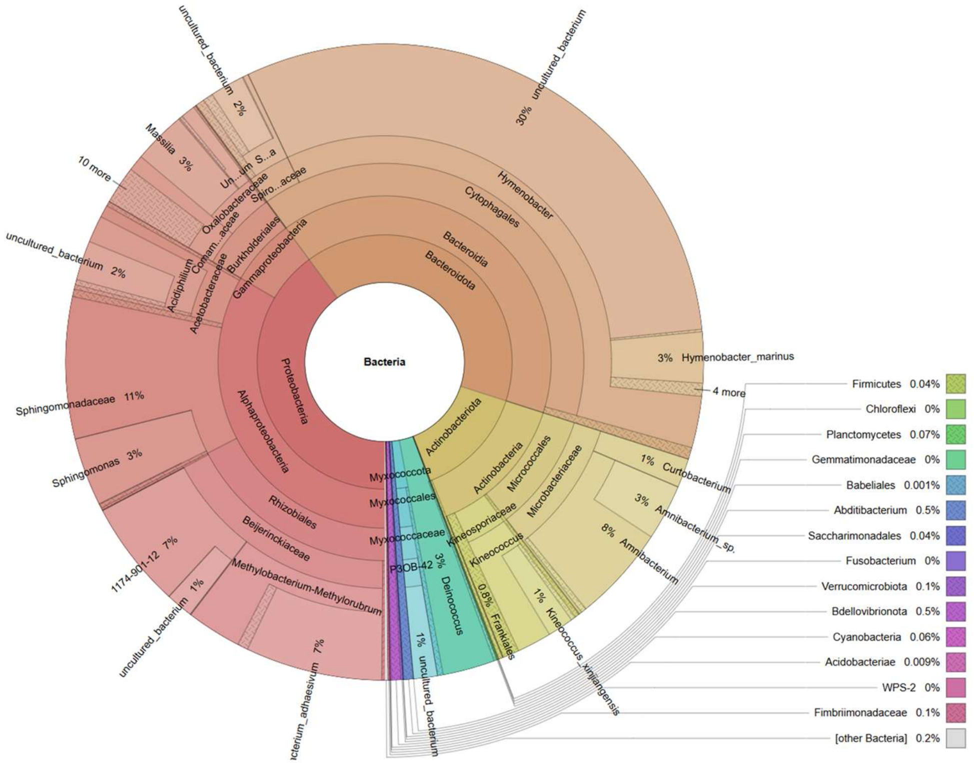

Figure S4 Phyllosphere bacterial community structure of holm oak leaves at T0

S2.4 Pyrene and chrysene deuterated metabolites produced in mesocosm experiments

<table>
<tr><th></th><th>OH-pyr-D9</th><th>OH-chr-D11</th></tr>
<tr><td>WCS-PhMi</td><td>Not detected in this mesocosm</td><td>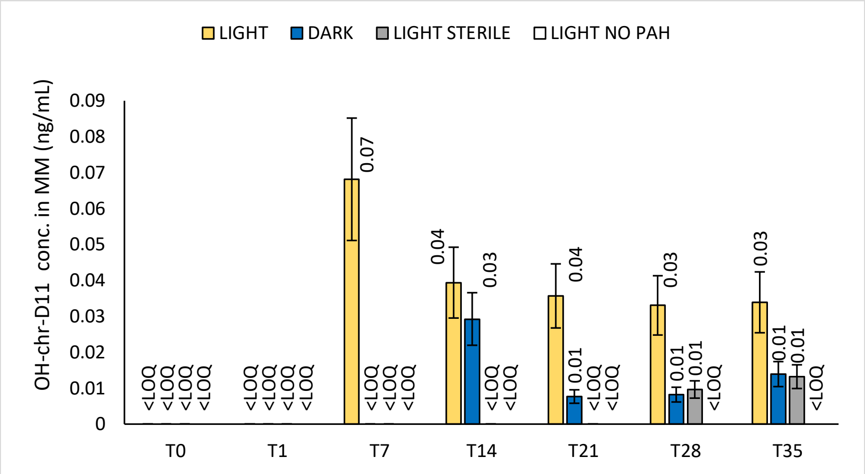
</td></tr>
<tr><td>WCS-EL</td><td>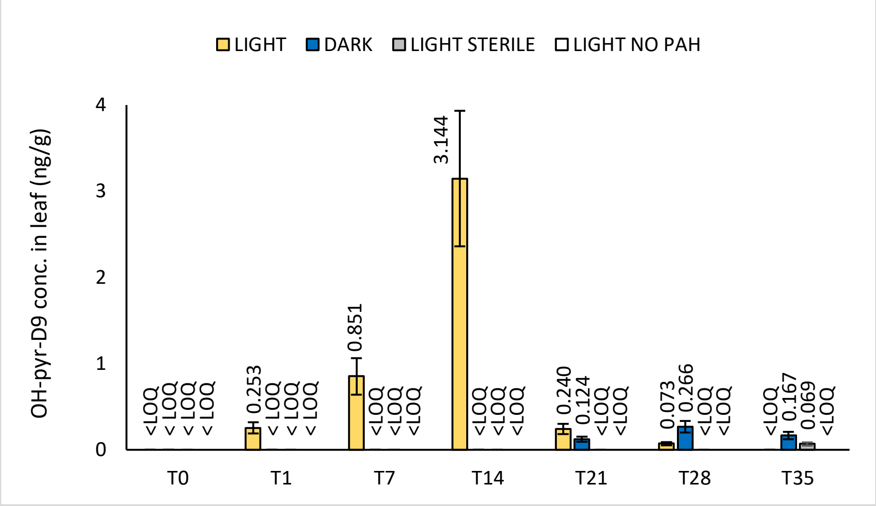
</td><td>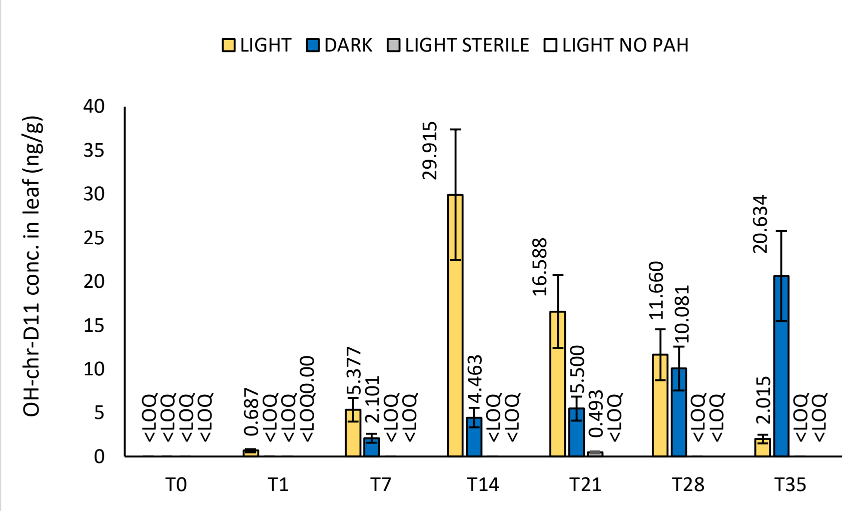
</td></tr>
</table>

Figure S5 Deuterated OH-pyrene (OH-PYR-D9) and OH-chrysene (OH-CHR-D11) concentrations produced in the mesocosm experiments (error bars (CV%) represent the analytical variability)

S2.5 Metabolite isomers produced in mesocosm experiments and in field

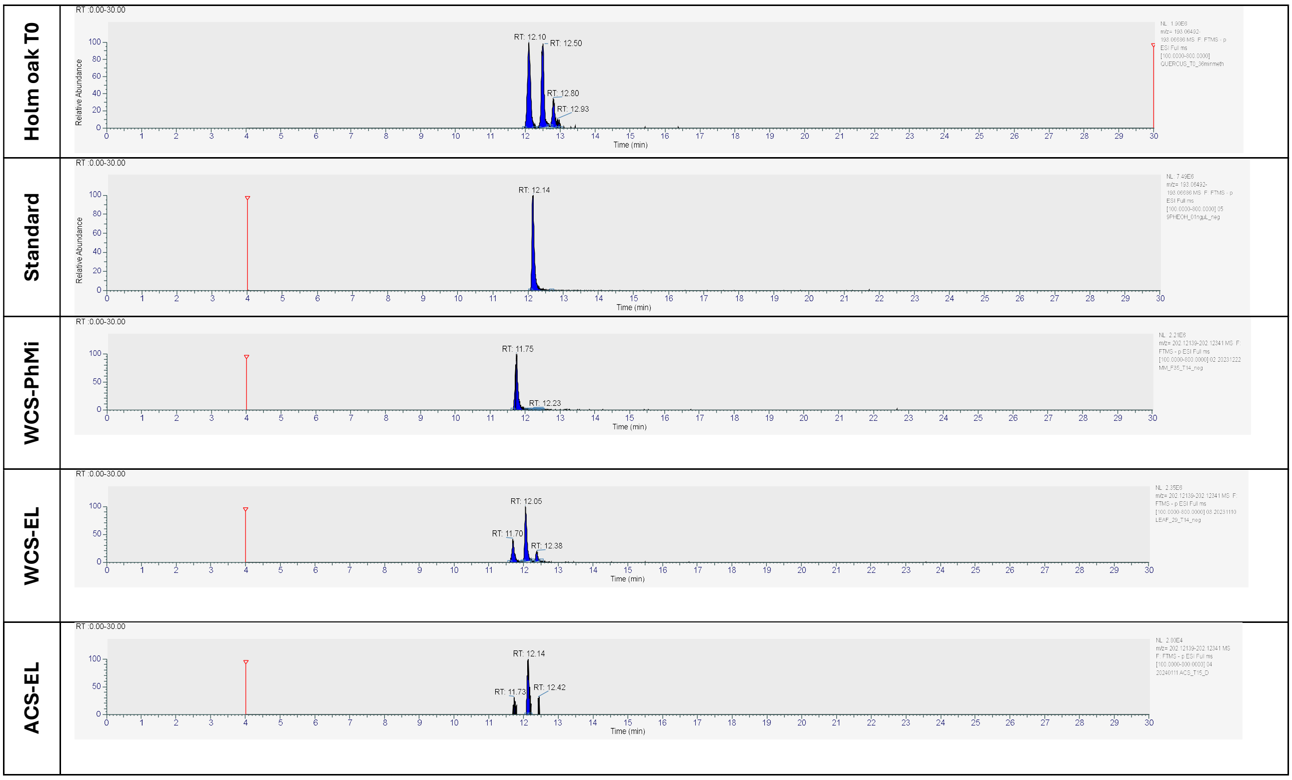

Figure S6 OH-PHE isomers (native for field and deuterated for laboratory experiments). RT of analytical standard (9-hydroxy-phenanthrene) is 12.14 min.

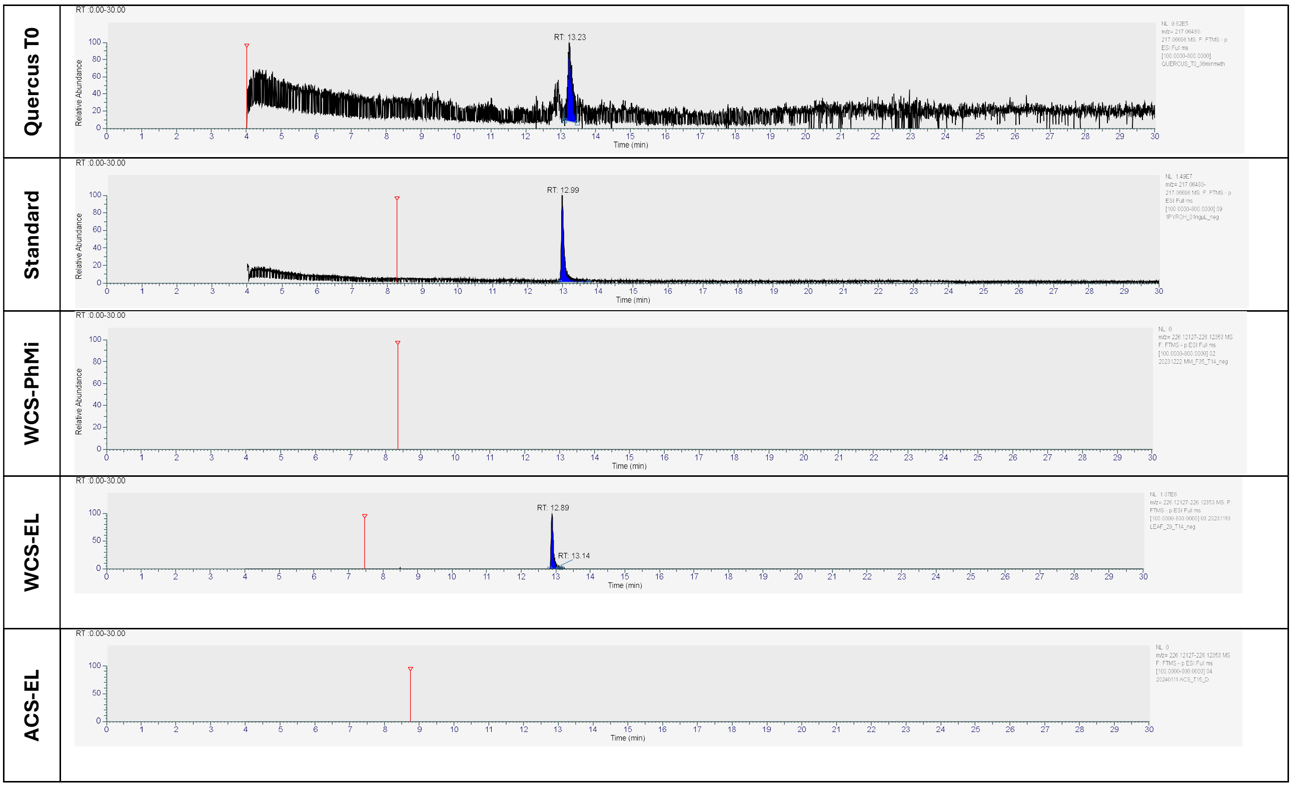

Figure S7 OH-PYR isomers (native for field and deuterated for laboratory experiments). RT of analytical standard (1-hydroxy-pyrene) is 12.99 min.

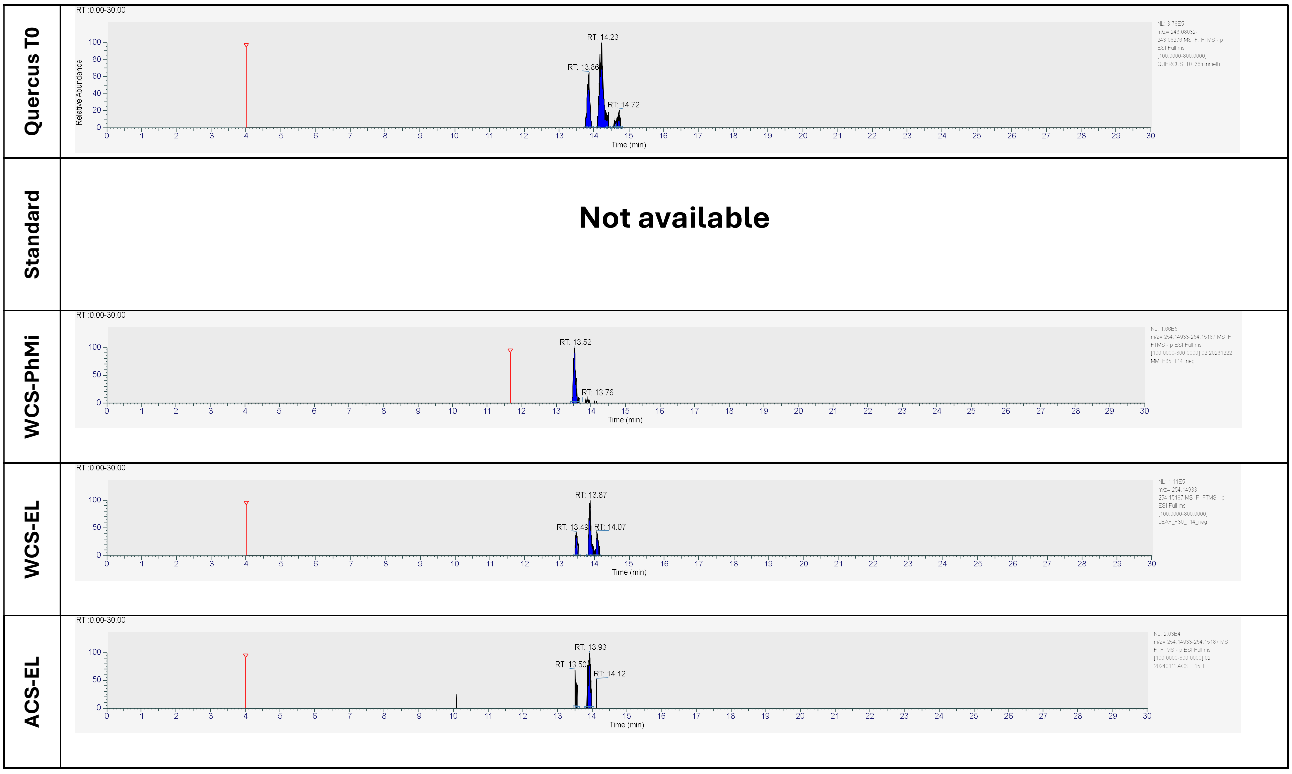

Figure S8 OH-CHR isomers (native for field and deuterated for laboratory experiments). Analytical standard for hydroxy-chrysene was not available.

S2.6 Bacterial community structure analysis in microcosm experiments

Table S6 Denoising and ASVs clustering statistics

Treatments	raw read pairs	primers-trimmed read pairs	filtered	percentage of input passed filter	denoised	merged	percentage of input merged	non-chimeric	percentage of input non-chimeric
**WCS-PhMi-S-T35**	321272	318757	259769	81.49	259474	257517	80.79	227082	71.24
**WCS-EL-D-T35**	310195	307620	242277	78.76	240314	233720	75.98	186844	60.74
**WCS-PhMi-D-T35**	302222	299918	245098	81.72	244192	240843	80.3	209451	69.84
**WCS-EL-L-T35**	263452	261477	210915	80.66	208265	198428	75.89	144270	55.18
**WCS-EL-S-T35**	240095	238025	191563	80.48	190538	187440	78.75	171157	71.91
**WCS-PhMi-L-T35**	232117	230352	185161	80.38	184057	181285	78.7	161318	70.03
**QUERCUS-T0-T35**	223320	221581	177002	79.88	174645	168120	75.87	144398	65.17

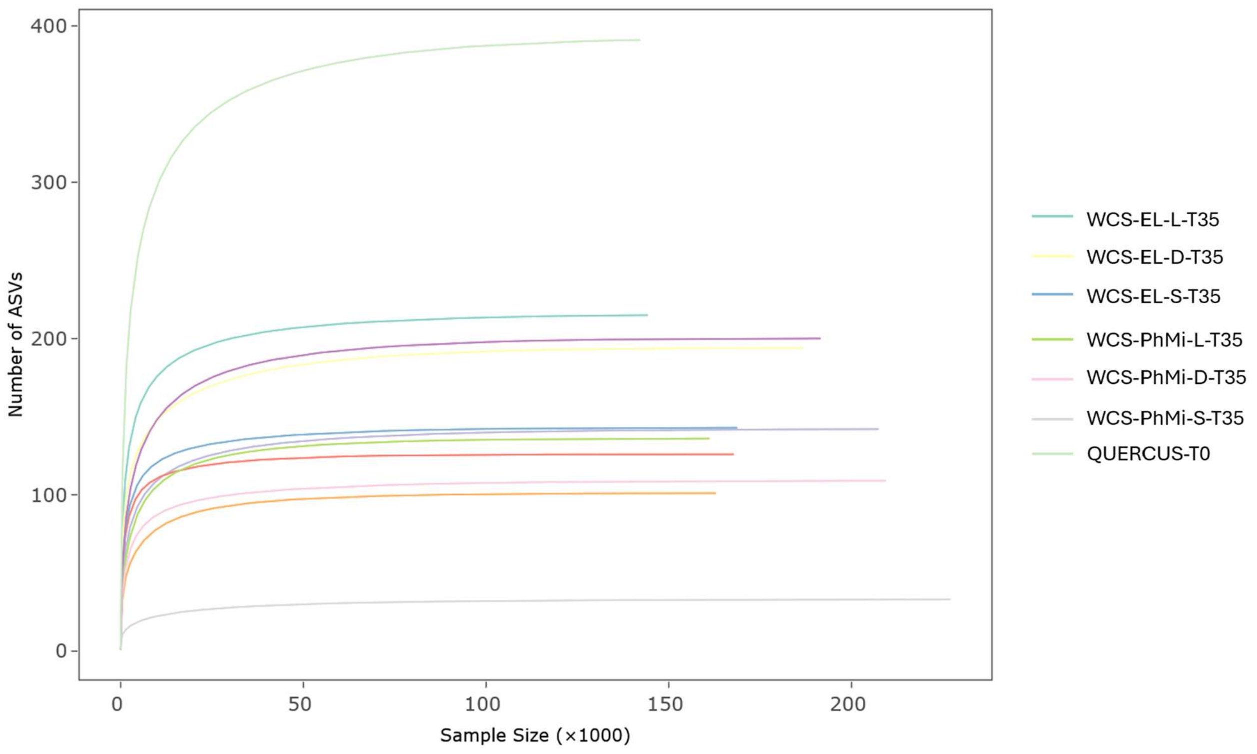

Figure S9 Rarefaction curves (bacterial diversity was optimally covered in all samples)

Table S7 Alpha-diversity indices

Treatments	Reads	Observed ASVs	Good's coverage	Chao1	Shannon	Simpson
**QUERCUS-T0**	142190	391	100	391.59	4.33	0.97
**WCS-EL-L-T35**	144270	215	100	215.11	3.5	0.93
**WCS-EL-D-T35**	186844	194	100	194	3.61	0.95
**WCS-EL-S-T35**	168772	143	100	143	3.03	0.9
**WCS-PhMi-L-T35**	161175	136	100	136	2.86	0.91
**WCS-PhMi-D-T35**	209451	109	100	109	2.07	0.75
**WCS-PhMi-S-T35**	227082	33	100	33	1.35	0.68

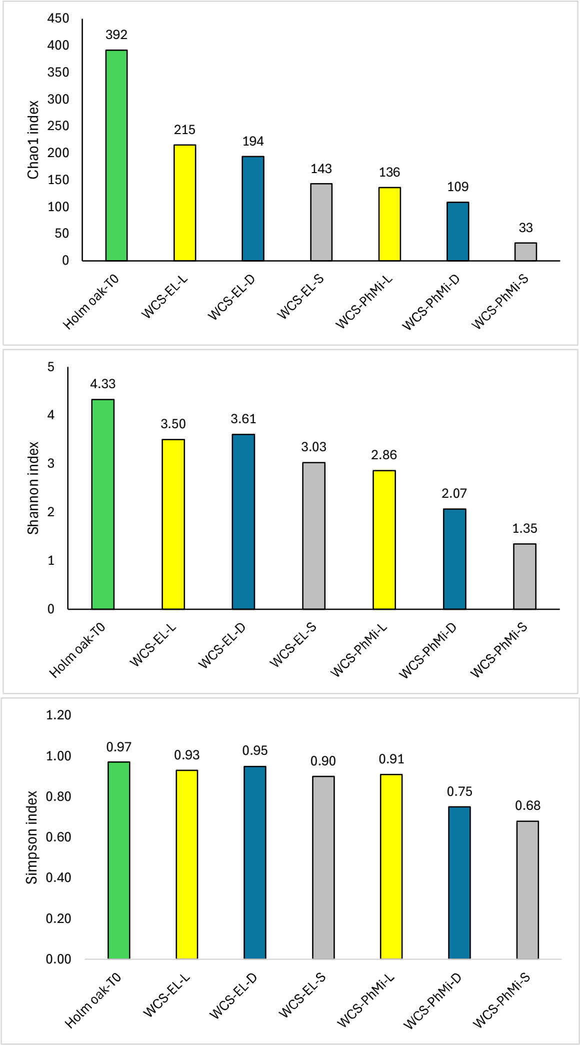

Figure S10 Alpha-diversity indices

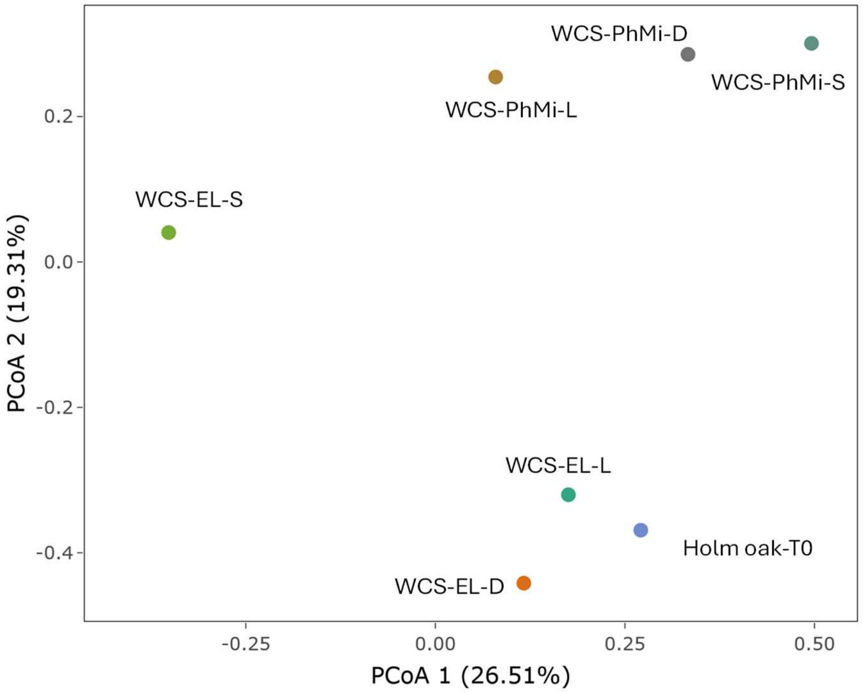

Figure S11 PCoA of Beta-diversity

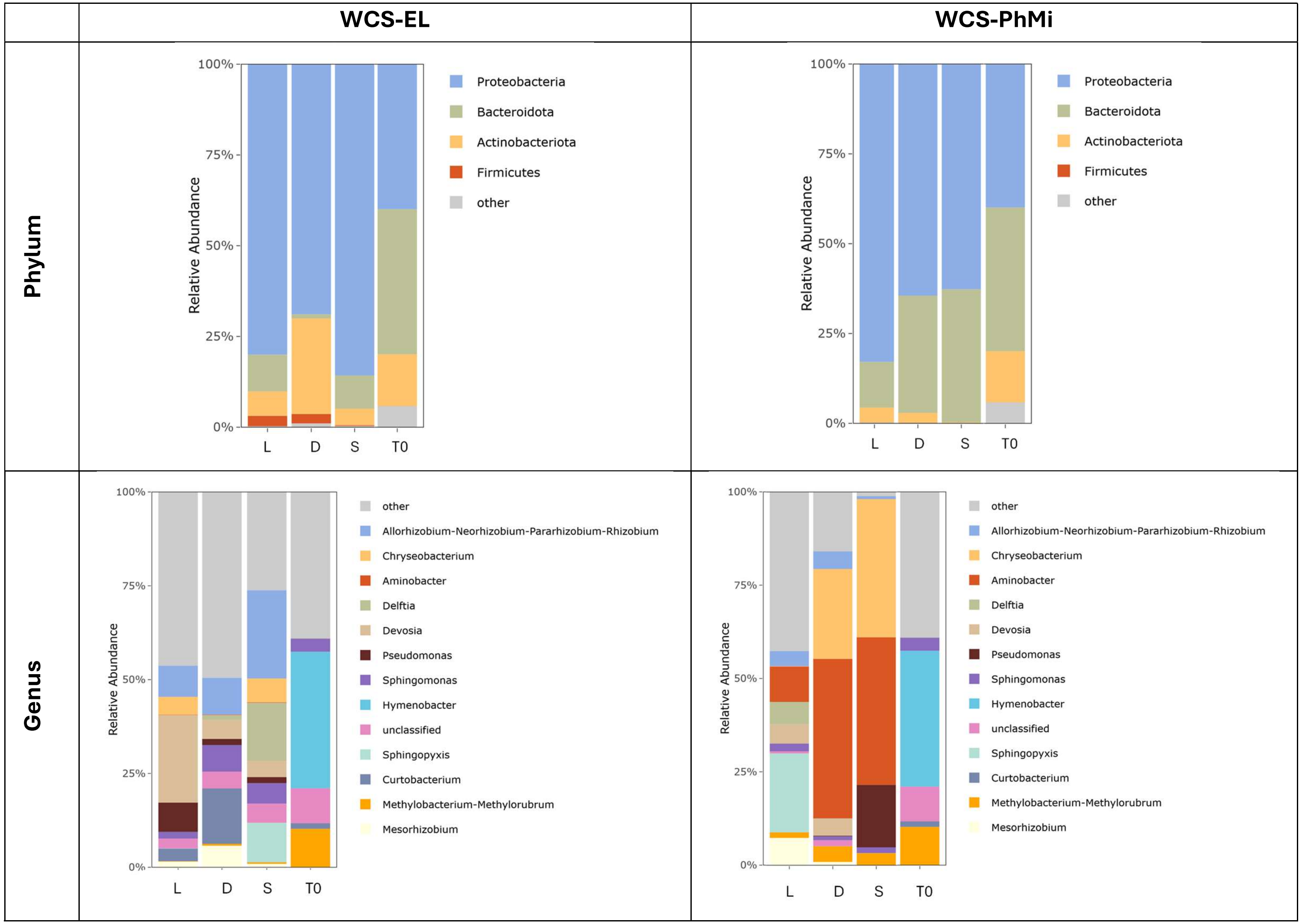

Figure S12 Comparison of microbial community (Phylum and Genus level) structure at T35 of each treatment of WCS-EL and WCS-PhMi system and holm oak leaves at T0. Letters under the bars refer to treatments: L is "Light", D is "dark", S is "Sterile", and "T0" is the native community at T0

REFERENCES

Bolyen, Evan, Jai Ram Rideout, Matthew R. Dillon, Nicholas A. Bokulich, Christian C. Abnet, Gabriel A. Al-Ghalith, Harriet Alexander, et al. 2019. “Reproducible, Interactive, Scalable and Extensible Microbiome Data Science Using QIIME 2.” Nature Biotechnology 37 (8): 852–57. https://doi.org/10.1038/s41587-019-0209-9.

Callahan, Benjamin J., Paul J. McMurdie, Michael J. Rosen, Andrew W. Han, Amy Jo A. Johnson, and Susan P. Holmes. 2016. “DADA2: High-Resolution Sample Inference from Illumina Amplicon Data.” Nature Methods 13 (7): 581. https://doi.org/10.1038/nmeth.3869.

Marques, M., Maitre, A., Choisnard, L., Demeilliers, C., Persoons, R., 2021. Simultaneous analysis of PAH urinary mono- and dihydroxylated metabolites by GC-MS-MS following SPE and two-stage derivatization. Anal. Bioanal. Chem. 413, 6823–6835. https://doi.org/10.1007/s00216-021-03638-4

Oksanen, Jari, Gavin L. Simpson, F. Guillaume Blanchet, Roeland Kindt, Pierre Legendre, Peter R. Minchin, R. B. O’Hara, et al. 2022. “Vegan: Community Ecology Package.” https://CRAN.R-project.org/package=vegan.

R Core Team. 2023. R: A Language and Environment for Statistical Computing. Vienna, Austria: R Foundation for Statistical Computing. https://www.R-project.org/.

Schindelin, J., Arganda-Carreras, I., Frise, E., Kaynig, V., Longair, M., Pietzsch, T., Preibisch, S., Rueden, C., Saalfeld, S., Schmid, B., Tinevez, J.-Y., White, D.J., Hartenstein, V., Eliceiri, K., Tomancak, P., Cardona, A., 2012. Fiji: an open-source platform for biological-image analysis. Nat. Methods 9, 676–682. https://doi.org/10.1038/nmeth.2019

Terzaghi, E., Wild, E., Zacchello, G., Cerabolini, B.E.L., Jones, K.C., Di Guardo, A., 2013. Forest Filter Effect: Role of leaves in capturing/releasing air particulate matter and its associated PAHs. Atmos. Environ. 74, 378–384. https://doi.org/10.1016/j.atmosenv.2013.04.013

US EPA, 1996. MEthod 3630C: Silica gel Cleanup.

US EPA, 1994. Method 3640A: Gel-Permeation Cleanup.
